# Hydroclimatic extremes threaten groundwater quality and stability

**DOI:** 10.1038/s41467-025-55890-2

**Published:** 2025-01-16

**Authors:** Simon A. Schroeter, Alice May Orme, Katharina Lehmann, Robert Lehmann, Narendrakumar M. Chaudhari, Kirsten Küsel, He Wang, Anke Hildebrandt, Kai Uwe Totsche, Susan Trumbore, Gerd Gleixner

**Affiliations:** 1 Department of Biogeochemical Processes, Max Planck Institute for Biogeochemistry, Jena, Germany; 2 Department of Hydrogeology, Institute of Geosciences, Friedrich Schiller University, Jena, Germany; 3 Aquatic Geomicrobiology, Institute of Biodiversity, Friedrich Schiller University, Jena, Germany; 4 German Centre for Integrative Biodiversity Research (iDiv) Halle-Jena-Leipzig, Leipzig, Germany; 5 Balance of the Microverse, Cluster of Excellence, Friedrich Schiller University, Jena, Germany; 6 Department Computational Hydrosystems, Helmholtz-Centre for Environmental Science – UFZ, Leipzig, Germany; 7 Terrestrial Ecohydrology, Institute of Geosciences, Friedrich Schiller University, Jena, Germany

**Keywords:** Biogeochemistry, Hydrology

## Abstract

Heavy precipitation, drought, and other hydroclimatic extremes occur more frequently than in the past climate reference period (1961–1990). Given their strong effect on groundwater recharge dynamics, these phenomena increase the vulnerability of groundwater quantity and quality. Over the course of the past decade, we have documented changes in the composition of dissolved organic matter in groundwater. We show that fractions of ingressing surface-derived organic molecules increased significantly as groundwater levels declined, whereas concentrations of dissolved organic carbon remained constant. Molecular composition changeover was accelerated following 2018’s extreme summer drought. These findings demonstrate that hydroclimatic extremes promote rapid transport between surface ecosystems and groundwaters, thereby enabling xenobiotic substances to evade microbial processing, accrue in greater abundance in groundwater, and potentially compromise the safe nature of these potable water sources. Groundwater quality is far more vulnerable to the impact of recent climate anomalies than is currently recognized, and the molecular composition of dissolved organic matter can be used as a comprehensive indicator for groundwater quality deterioration.

## Abstract (web summary)

This molecular-level study provides a comprehensive assessment of groundwater quality in three German research sites. Hydroclimatic variability is causing fundamental changes in groundwater ecosystems, with implications for global water resources.

## Introduction

As billions of people rely on groundwater as a primary source of drinking water^1^, the continuous availability, cleanliness, and safety of this resource is imperative^2^. The availability of valuable clean groundwater is threatened by increasingly extreme hydroclimatic conditions^3–7^ that alter the functioning of natural recharging processes^8–13^. Extreme hydroclimatic conditions such as extended drought periods or lasting heavy precipitation events are known to have severe and complicated consequences on the biogeochemical processing, the generation of mobile matter, and the hydraulic structure in soils and the vadose zone^14,15^. In sum, greater seepage volumes with greater matter loads are translocated more rapidly along preferential flow paths downwards, thereby escaping retention in soil and the vadose zone. Recent investigations warn that the deleterious effect of hydroclimatic variations on natural groundwater quality could exceed that of anthropogenic pollution^16^. Thus, drinking water from groundwater sources could rapidly become less reliable^10,17–19^. We hypothesize that the efficiencies of natural filtering and transformative processes that render groundwater clean and safe to drink are declining as a result of increasingly common hydroclimatic extremes^20–22^.

Dissolved organic matter (DOM), a highly complex mixture of thousands of natural substances records the collective action of seemingly countless biogeochemical processes that purify water as it percolates through the subsurface^23–27^. The mediating role of DOM in the mineral sorption of aromatic substances leaching from plant litter and soil as well as their active transformation by microbial communities to cellular necromass could be shown via molecular-level analyses based on direct-infusion ultrahigh-resolution mass spectrometry (DI-HR-MS)^28–31^. Recent groundwater studies demonstrated that DOM DI-HR-MS data could inform about aquifer hydrogeology^32^, microbial community health^24,33^, and groundwater quality/recharge dynamics^34^. DI-HR-MS does not rely on chromatographic separation, but on the great mass resolution provided by Fourier-transform ion cyclotron resonance or, here, Orbitrap analyzers. Resulting data reveal relative abundances of thousands of distinct molecular entities. Relative abundance patterns of the same sample type can differ between instrument types and change with method parameters^35^. Knowing this, we used a single method and measured repeated standards to be able to demonstrate the scalability of DI-HR-MS analyses for long-term and large-scale efforts aimed at exploiting DOM molecular composition data to infer groundwater quality under variable and extreme conditions.

Extensive infrastructure is required to monitor the natural processing of water from the surface to the subsurface. This includes soil seepage samplers^14^ and groundwater wells in various hydrogeological settings, both of which are to be managed by state-of-the-art protocols and instrumentation for standardized long-term sampling and analysis^36^. Considering time series of up to 8 years, and comprising 254 groundwater and 268 soil seepage DOM DI-HR-MS analyses, the findings of this investigation provide one of the most comprehensive molecular-level assessments of natural groundwater quality to date.

The overarching goal of this study is to leverage the extreme molecular complexity of DOM and provide a simple and scalable metric that is more sensitive to changes in natural groundwater purification efficiency than the commonly used bulk concentration of groundwater DOC. We base our assessment of groundwater purification efficiency on the molecular-level Bray-Curtis similarities of groundwater and soil seepage DOM under changing hydroclimatic conditions between 2014 and 2021 at three geologically distinct Critical Zone research sites in Germany^37,38^. Support for the interpretation of changes in DOM composition is provided by groundwater stable isotope ratios and DOM radiocarbon isotope ratios, which track groundwater infiltration and the transport of ^14^C-young, surface-derived substances, respectively. We omit common assumptions about indicative compound classes and instead link DOM to metabolic functions recorded in groundwater metagenomes. Resulting data strongly suggest that hydroclimatic variability causes fundamental changes in natural groundwater quality, and its impact is likely to intensify the global groundwater crisis^39,40^.

## Results and discussion

### Increasingly variable groundwater infiltration

**Fig. 1:**
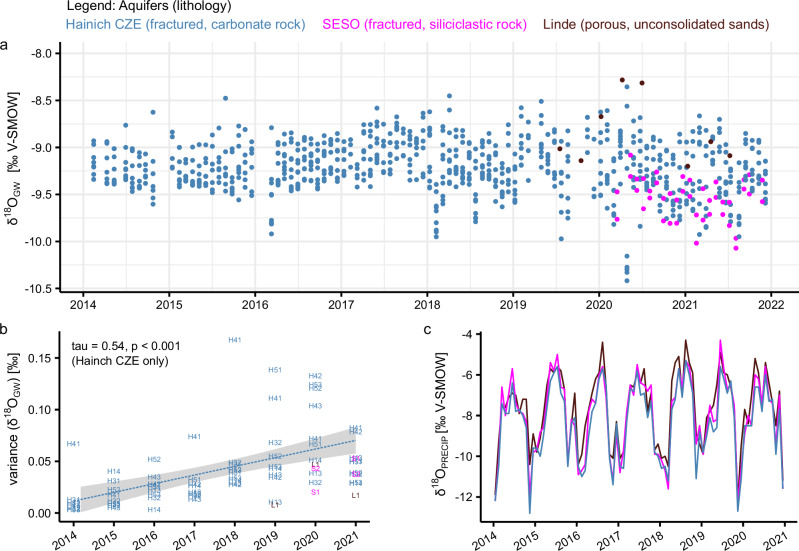
Stable isotope ratios (δ^18^O) in groundwater (*n* = 839) and precipitation. **a** Groundwater stable isotope ratio ranges reflect mixtures of summer and winter precipitation sans change to the mean over time. Time series suggest increasing variability in stable isotope ratios after the extreme 2018 summer drought in Europe. **b** Variances in stable isotope ratios per groundwater well and year. Significant linear increases in intra-annual variances over a period of eight years at Hainich Critical Zone Exploratory (CZE) show that episodic and event-based transport are increasingly prominent determinants of groundwater recharge dynamics. The dashed line represents a linear regression and 95% confidence interval. Significance was evaluated using Kendall’s rank correlation tau. During the extreme drought year of 2018, a nonlinear increase in variance is revealed for well H41. This well has been previously shown to respond very rapidly and strongly to recharge and recession dynamics^34^. This suggests that in addition to the linear long-term increase in infiltration variability, groundwaters are locally highly vulnerable to hydroclimatic extremes on short-time scales. **c** Seasonal variability in stable isotope ratios of precipitation from PisoAI^86^. Figure created in R.4.4.0 using package *ggplot2*.

In the aquifer regions studied, 12 out of 13 studied groundwater wells showed consistent declines in hydraulic heads, ranging from −0.7 to −106 cm per year between 2014 and 2021 (Supplementary Fig. 1). A general trend is observed during this period both across Germany^11^ and globally^13,41^ that can reflect both climate change and increased withdrawal. As a result, a significant loss of global groundwater reserves is expected by the end of the century. At our sites, declines correspond to decreases in annual precipitation that are reflected in increasing dryness across Europe^42^ (Supplementary Fig. 2). At the Hainich Critical Zone Exploratory (CZE), heavy rainfall fluxes (>10 mm/day) remained close to constant, whereas lighter precipitation significantly decreased, matching recent findings of more extreme precipitation dynamics across Central Europe^3^. Correspondingly, episodes of intense groundwater infiltration fluxes have become an increasingly important component of groundwater recharge, as indicated by increases in intra-annual variances of groundwater stable isotopes (Fig. 1a, b). Stable isotope ratios of precipitation carry a seasonally varying signal into the aquifers, where many contributing water sources (temporal and spatial) accumulate and mix^43^ (Fig. 1c). The likelihood of groundwater infiltration fluxes to be able to affect and sway the stable isotope ratio of the local groundwater bodies is expected to be greater following heavy short-term rainfall than light rainfall more spread out over the season. Episodic soil-to-groundwater fluxes can also transport large amounts of natural organic matter deep into the subsurface, thereby threatening the otherwise high natural purity of shallow groundwater resources^14,27,34,44^. Therefore, it is prudent to determine the extent to which groundwater purity was altered and its vulnerability to future episodic and/or event-based infiltration.

### Altered soil-to-groundwater transfer

**Fig. 2:**
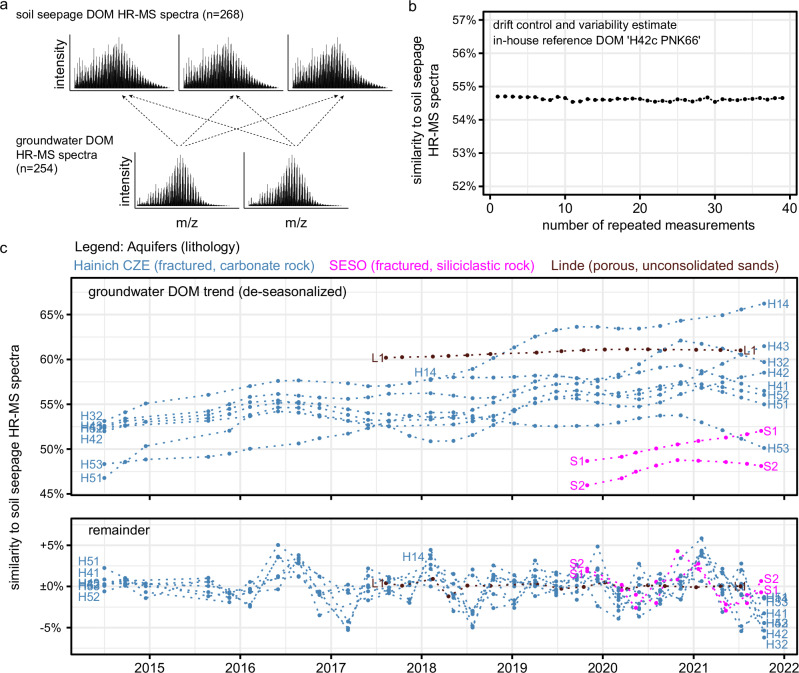
Seasonal time series decomposition of molecular similarities of groundwater (*n* = 254) to soil seepage dissolved organic matter (DOM, *n* = 268). **a** Overview of DI-HR-MS spectra of soil seepage and groundwater DOM. Arrows indicate that calculated percentage similarities (PS) reference each groundwater sample to all local soil seepage samples to account for soil heterogeneity. These are reported as averages in panel (**c**). The standard deviation among PS values of soil seepage DOM was 5%. **b** Drift control and measurement variability estimate based on repeated measurements of an in-house groundwater DOM reference (*n* = 39) interspersed with the entire sequence of measurements. The standard deviation of PS values in the reference measurements was 0.05 percentage points, with no drift being observed. **c** Consistent long-term trend towards greater contributions of soil seepage-derived substances to groundwater DOM. This suggests a reduction in the efficiency of natural processes by which water is purified during percolation into aquifers. Groundwater well depths and screen lengths are available in Supplementary Fig. 1. Figure created in R.4.4.0 using package *ggplot2*.

All wells that significantly decreased in hydraulic head also showed increasing amounts of soil seepage-derived substances, based on a seasonal time series decomposition (STL) of similarities between groundwater and soil seepage DOM DI-HR-MS spectra (Fig. 2a). Between 2014 and 2021, groundwater DOM at the Hainich CZE has become more than 10 per cent more similar to soil seepage DOM (Fig. 2c). The three- and five-year time series at SESO and Linde revealed changes of five and one per cent, respectively. These effect sizes reflect significant changes in natural groundwater quality (*p* < 0.001), considering a standard deviation of 0.05 percentage points based on the same calculations performed on repeated measurements (*n* = 39) of an in-house groundwater DOM standard interspersed throughout the entire sequence of DOM DI-HR-MS measurements (Fig. 2b). Long-term trends reflect an intensification of the soil-to-groundwater connection, coupled to a reduction in efficiency of natural filtrations and purifications that occur during percolation from the vadose zone into the aquifers^28^. Groundwater in fracture-flow aquifer systems appeared markedly more vulnerable than groundwater in porous, unconsolidated sands, considering the rates of DOM composition change being an order of magnitude lower at the Linde site compared to Hainich CZE and SESO (Supplementary Table 1). This highlights the importance of preferential flow paths along fractures in the subsurface, allowing surface-derived substances to evade purification processes during rapid groundwater infiltration. The role of fractures in providing preferential flow paths is even more important for marine bedrock like those of the Hainich CZE because of their matrix tightness (low porosity and permeability)^45^.

Our results suggest that by leveraging its extremely diverse composition, DOM can be exploited as an early indicator of the deterioration of groundwater quality. DI-HR-MS revealed a significant increase in soil seepage-sourced contributions to groundwater DOM, whereas the commonly monitored concentration of DOC did not show significant changes during the observation period (Supplementary Fig. 3). It is likely that the previously shown great bioavailability of surface-derived DOC in groundwater contributed to the stability of groundwater DOC^46^. The change in DOM composition would thus be expected to be driven rather by microbial metabolites and surface-derived substances, which are not readily biodegradable and are not individually highly abundant. Measurements of the radioactive ^14^C isotope on the same groundwater DOM extracts analyzed via DI-HR-MS provided independent validation of the interpretation that changes in groundwater DOM composition were being driven by surface-derived inputs. We observed a positive correlation (*p* < 0.001) between the fraction of modern carbon (F^14^C) in groundwater DOM and the calculated similarities between groundwater and soil seepage HR-MS spectra (Supplementary Fig. 4). The correlation was not heavily driven by low F^14^C values in the anoxic wells H42, H43, H52, and H53 and was also significant (*p* < 0.001) when considering only oxic groundwater from the Hainich CZE^34^. This finding underscored the potential of DOM DI-HR-MS data as meaningful proxies by which to accurately appraise the impact(s) of soil seepage-sourced contributions to groundwater ecosystems.

The rate of year-on-year changeover in groundwater DOM composition was greater following the extreme summer drought of 2018 compared to the preceding period, based on respective linear regressions of the similarity values shown in Fig. 2c (Supplementary Table 1). Seven of the eight wells with continuous long-term data showed steeper changes in their similarity to soil seepage HR-MS spectra after July 2018, with the only exception being well H53, which was previously shown to be barely affected by surface-derived matter fluxes^34,44^. In 2018 and 2019, central Europe experienced significant summer droughts, each of which had devastating ecological consequences and represented an extreme hydroclimatic anomaly made more likely by anthropogenic-induced global warming^4,5,47–49^. During and following the summer of 2018, our investigation sites faced the greatest local annual water deficits within the period 2006–2021 (Supplementary Fig. 5). These were accompanied by steep declines in hydraulic heads following previous groundwater highstands in early 2018 in the Hainich CZE (Supplementary Fig. 1). Our observations suggest that extreme hydroclimatic conditions could substantially alter groundwater recharge dynamics, and in turn, drive groundwater quality decline.

In a previous multi-year investigation of soil seepage at the Linde site it was found that following summer dryness DOM was rich in plant-derived aromatic substances that were not yet microbially processed^50^. It is likely that upon initial precipitation and rewetting events, pulses of unprocessed DOM bypassed Critical Zone filtration and purification processing and proceeded directly to aquifers via preferential flow paths^51^. Preferential soil-to-groundwater DOM transport could overload natural filtering and transformation processes, thereby driving long-lasting change in aquifer ecosystems^34,52^. In addition, the fraction of microbial taxa transported via seepage to the groundwater microbiome roughly doubled in 2018, compared to the long-term mean^53^. Given the vast potential of chemical transformations inherent in healthy microbial communities, the joint transport of soil seepage-derived DOM and bacteria following the hydroclimatic extreme of 2018 potentially fundamentally altered natural groundwater purification. The data collected in this study clearly demonstrate that climate change-induced extreme hydroclimatic conditions affect groundwater quality.

### Implications for groundwater ecosystems

**Fig. 3:**
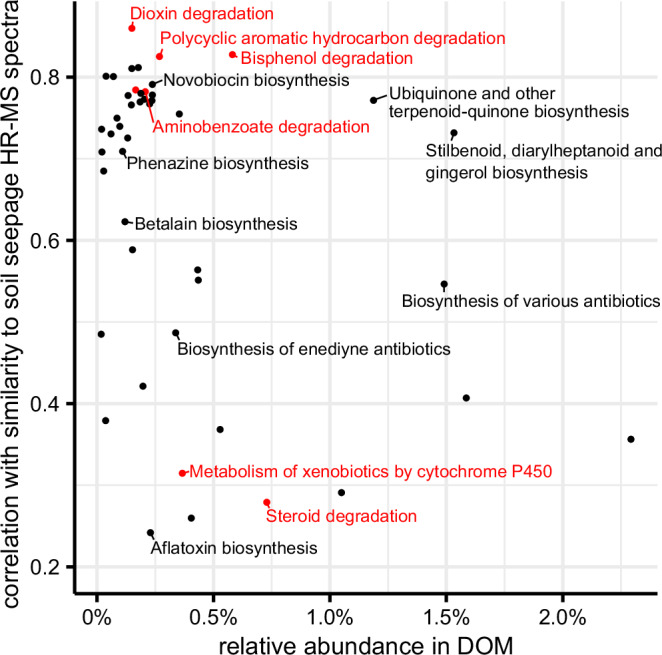
Metabolic pathway prediction via the KEGG database based on groundwater direct-infusion high-resolution mass spectrometry (DI-HR-MS) molecular formulas constrained by metagenome-assembled genomes from the same well(s); *n* = 254 for DI-HR-MS, *n* = 32 for groundwater metagenomes. Each pathway shown has ≥10 molecular formulas or ≥25% of its theoretical total length being detected. Pathways depicted show a significant positive correlation with the percentage similarity of groundwater to soil seepage dissolved organic matter presented in Fig. 2 (*p* < 0.01, Bonferroni corrected). Pathways belonging to the KEGG pathway group “Xenobiotics biodegradation and biosynthesis” are colored in red. Other pathways relating to the biosynthesis of natural antibiotics and toxins, as well as abundant pathways, are named in black text. Detailed information on the pathway prediction workflow is available in Supplementary Fig. 6. Figure created in R.4.4.0 using package *ggplot2*.

Results suggest that the observed increase in the similarity of groundwater to soil seepage DOM HR-MS spectra affected several metabolic functions of the groundwater microbiome. We directly compared the similarity scores reported in Fig. 2 with the relative abundances of predicted molecular formulas in groundwater, summed by metabolic pathways via a matching to the KEGG database. A dereplicated, quality-controlled data set of 1224 metagenome-assembled genomes (MAGs) obtained from the same wells of the Hainich CZE was used to constrain metabolic pathway predictions from DOM to the functional potential of groundwater microbial communities^54^. 717 molecular formulas (10%) out of 7277 matched to metabolic functions of the groundwater microbiome. In spite of some sum formulas having multiple isomers, more than 70% of sum formulas could be unambiguously linked to specific metabolic pathways (Supplementary Figs. 6,  7). Rank correlations between summed relative abundances of sum formulas per metabolic pathways and calculated similarities to soil seepage HR-MS spectra are shown in Fig. 3. Each dot represents a distinct metabolic pathway, and its y-axis position indicates how strongly the pathway’s relative abundance pattern correlated to observed increases in similarity of groundwater to soil seepage DOM HR-MS spectra over the same set of samples. Many of the most strongly correlated pathways are involved in xenobiotics metabolism (highlighted in red) or the biosynthesis of natural antibiotics and toxins. Strong responses of xenobiotic and aromatic hydrocarbon breakdown pathways in groundwater DOM suggest that during rapid and episodic groundwater recharge, these substances evade retention and microbial processing in the (sub)soil^52,55,56^. Metabolic pathways related to the biosynthesis of toxins and natural antibiotics (e.g., novobiocin, prodigiosin, aflatoxin biosynthesis) likely originate from bacterial-bacterial and bacterial-fungal antagonism in the topsoil^57–59^. Our findings emphasize the high potential of DOM DI-HR-MS analyses to warn about important ongoing changes in natural groundwater quality before threshold levels of harmful environmental xenobiotics^60^ or bulk DOC concentration could be reached. As climate change effects intensify, this trend could portend larger, rapid changes in the functioning of groundwater ecosystems, calling into question the future security of essential ecosystem assets^24,61–63^.

As the number of extreme precipitation and drought events in central Europe has significantly increased^3,5,11,47^, groundwater infiltration has become much more punctuated and variable. Collectively, the findings of our investigation demonstrate that irregular transitioning of waters from soils to aquifers adversely affects both groundwater quantity and quality. Based on the rate of change observed in the period 2014–2021, the vulnerability of naturally purified groundwater resources is likely to increase, amplifying the stress society already faces concerning diminishing groundwater levels^41,64^.

Studying and monitoring groundwater quality via a DOM proxy affords a unique opportunity to continuously assess the natural vulnerability of these precious ecosystems to hydroclimatic extremes. This capability is particularly germane to locations where anthropogenic contamination is hitherto limited, and groundwaters are, without any empirical support, thought to be clean and safe for drinking well into the future. Based on studied lithologies and aquifer properties as well as forecasts of the European hydroclimate, important regional aquifers featuring topographic recharge, e.g., in central Italy, northeastern Spain, central and southwestern France, as well as throughout Germany, could potentially face trends similar to our reported findings^7,38,42^.

Current groundwater quality typically uses only the bulk concentration of DOC^65,66^. We suggest that the availability of high-resolution molecular composition data of DOM could evaluate patterns that provide a clearer indication of groundwater quality deterioration. Timely action could prove critical, as climate and environmental change already disturb the homeostatic functioning of the Critical Zone, thereby deleteriously affecting groundwater availability and safety on a much larger scale and at a much faster rate than is currently recognized^67,68^.

## Methods

### Study sites

We studied three hydrogeologically distinct aquifer systems in central and northeastern Germany, encompassing fractured-karstic carbonate rocks of the Hainich Critical Zone Exploratory (CZE), fractured-porous siliciclastic rocks of the Saale-Elster-Sandsteinplatte Observatory (SESO) and glacial sands at the Forschungsstation Linde. These sites were selected to represent major lithologies and aquifer properties dominant throughout Central Europe^37,38^. All sites were well studied, with previous investigations detailing the functioning of the entire Critical Zone. The Hainich CZE in northwest Thuringia (central Germany) represents a hillslope sub-catchment of used groundwater resources in a temperate climate. The Hainich CZE monitoring well transect accesses a fracture-flow aquifer system in sloping thin-bedded limestone-mudstone alternations. Aquifer compartments at the Hainich CZE differ strongly in oxygen availability. Wells H13, H14, H31, H32, H41, and H51 are oxic, whereas H42, H43, H52, and H53 are anoxic ( < 0.1 mg/L dissolved oxygen). Redox potentials range around 400 mV for wells H13, H14, H31, H32, H41, and H51, and around 200 mV for wells H42, H43, H52, and H53. The contribution areas for accessed groundwater in the Hainich CZE underlie forest, grasslands, and croplands. The SESO aquifer system is located in central Thuringia within fractured-porous siliciclastic bedrock. While the SESO differs from the Hainich CZE in hydrogeological structure and soil acidity, these sites share similar land-use types, vegetation, and topographic recharge in hillslope settings. Groundwater accessed at SESO is oxic. Forschungsstation Linde, located in Brandenburg (northeast Germany) garners access to an aquifer in non-sloping topography that has developed upon glacial sands and gravels beneath a diverse forest setting. Groundwater accessed at the Forschungsstation Linde is oxic. Thorough descriptions of the study sites are available in previous publications for the Hainich CZE^34,69^, SESO^70^, and Linde^25,56^. The depths of the respective groundwater screen sections are shown in Supplementary Fig. 1 and details on the setup of the groundwater wells are available in ref. ^69^. Fieldwork permits were issued by the responsible state environmental offices, local authorities and landowners.

### Sampling

Groundwater was extracted from the wells with submersible pumps (MP1 or SQE 5-70, Grundfos, Denmark) in monthly intervals. After steady physicochemical parameters were reached in flow-through cells, 100 mL of groundwater were sampled for DOC quantification in 100 mL borosilicate bottles with polypropylene caps (VWR, Darmstadt, Germany). Every third month, 10 L of groundwater were additionally sampled in duplicate HDPE canisters for DOM DI-HR-MS. Groundwater samples were immediately transported to the laboratory, filtered to <0.7 µm, acidified to pH = 2 with HCl, and stored at 4 °C in the dark until further processing. Soil seepage was collected from permanently installed tension-controlled lysimeters in fortnightly intervals based on water availability. Soil seepage samples were immediately transported to the laboratory and stored frozen at −20 °C in 250 mL polycarbonate bottles (Thermo Fisher Scientific, Dreieich, Germany) until filtration to <0.7 µm and further processing. Details on the setup of the lysimeters in mixed beech forest and grassland in Hainich CZE are available in ref. ^14^. Briefly, lysimeters in Hainich CZE were setup in 30 and 60-cm depth and featured porous silicon carbide suction plates (SIC320, mean pore size: 21 µm, thickness 10 mm, area 0.08 m²; METER, Vöhringen, Germany) installed below an undisturbed soil profile without sidewalls. Details of the setup in Beech, Oak, and Pine forests, as well as grassland at the Research Station Linde, are available in ref. ^50^. Briefly, sintered glass suction plates (pore size: 1–1.6 µm, thickness: 10 mm, area 0.045 m²; UMS, Willmars, Germany) were installed in Linde in 5, 10, 20, and 30 cm depth below an undisturbed soil profile. Seepage data from SESO unavailable. Calculations in Fig. 2 for SESO thus reference to seepage from Hainich CZE, as both vegetation and topography are highly similar between both sites.

### Groundwater stable isotopes

Stable hydrogen (δ^2^H) and oxygen (δ^18^O) isotopes of groundwater were analyzed as triplicate measurements at the MPI-BGC in Jena, Germany, using a Delta+ XL isotope ratio mass spectrometer (Finnigan MAT, Bremen, Germany). Measurements were calibrated in accordance with in-house standards, which were regularly verified against Vienna standard mean ocean water (VSMOW: δ^2^H = 0‰, δ^18^O = 0‰) and standard light antarctic precipitation (SLAP: δ^2^H = −428.0‰, δ^18^O = −55.5‰). A detailed explanation of the instrument and methodology is provided in ref. ^71^. Intra-annual variance was calculated in R4.2.1 using the stats::var() function^72^ as ${{\mathrm{var}}}=\sum {\left(x-\bar{x}\right)}^{2}/\left(n-1\right)$ with ($x-\bar{x}$) as the deviation from the mean and *n* as the number of observations. The mean-variance of technical triplicate measurements was 0.1‰.

### Molecular composition of DOM

DOM was extracted following a common solid-phase extraction (SPE) protocol using 5 g PPL Bond Elut cartridges (Agilent Technologies, Waldbronn, Germany) for groundwater and 1 g cartridges for soil seepage samples^73^. Groundwater samples were processed in duplicate. The volume passed over the PPL cartridges was adjusted based on the DOC concentration in the respective sample, quantified as non-purgeable organic carbon on a vario TOC cube (Elementar Analysensysteme, Langenselbold, Germany). This step was necessary only for the soil seepage samples due to their higher DOC concentration compared to groundwater. PPL is a nonpolar resin based on a styrene-divinylbenzene polymer that has been shown to yield high extraction efficiencies, high-quality molecular composition spectra, and clean procedural blanks, outperforming 23 other SPE materials in terms of overall representativeness in a previous investigation^74^. We note that, while necessary to obtain salt-free samples for electrospray ionization (ESI) mass spectrometry, any type of SPE pretreatment will be partially incomplete due to the specific properties of the adsorber material. In the case of PPL, the most likely compound classes to be underrepresented compared to the raw material are polar organic substances, such as amino acids and sugars. PPL extracts were kept at −80 °C in 10 mL amber borosilicate vials (CarlRoth, Karlsruhe, Germany) for long-term storage.

For direct-infusion HR-MS analyses, the concentration of extracts was adjusted to 20 mg C/L in a 1:1 water and methanol solvent mixture. Then, 100 µL of DOM extract was injected into a continuous flow of 20 µL/min water and methanol (1:1) using an UltiMate 3000 (Thermo Fisher Scientific, Waltham, USA). Measurements were recorded on an Orbitrap Elite mass spectrometer (Thermo Fisher Scientific, Waltham, USA) with a mass resolution of 555,000 ± 9000 at m/z = 251. ESI was run in negative ionization mode with an ESI needle voltage of 2.65 kV. For each sample, 100 scans of m/z 100–1000 were acquired and averaged. Instrument calibration was performed according to the vendor’s instructions using Pierce ESI Calibration Mixture for negative mode (Thermo Fisher Scientific, Waltham, USA).

After the acquisition, spectra were processed using DOMAssignR^75^, an open-source tool for standardized DOM mass spectra processing. DOMAssignR is a wrapper around MFAssignR^76^ and is available at https://github.com/simonschroeter/DOMAssignR. Details on DOM data processing are available in the code availability section. Briefly, we employed a signal-to-noise >10 filters and recalibrated the spectra to <1 ppm mass accuracy covering the entire m/z range from approx. m/z = 105 up to, at minimum, the m/z value where 80% of the cumulated intensity of the respective spectrum was reached (commonly approx. m/z = 600). The most abundant m/z values present in blanks (up to 95% cumulated intensity of the blanks) were not considered further. Only m/z values detected in both biological replicates were considered for groundwater samples. Spectra were normalized to the sum of all peak intensities. The percentage similarity (PS) between groundwater and soil seepage HR-MS DOM spectra was calculated as ${PS}=\left(1-\left({Bray\; Curtis\; dissimilarity}\right)\right)*100$ based on all m/z values above the S/N threshold. PS values for groundwater from the Linde site reference to soil seepage from Linde, those for groundwater from Hainich CZE and SESO reference to soil seepage from Hainich CZE. PS values were first calculated between all samples, and then averaged over all soil seepage sampling time points and local sampling locations to maximize robustness against surface heterogeneity. The reported changes in PS are thus solely being driven by changes in groundwater DOM composition. Seasonal time series decomposition by LOESS (locally estimated scatterplot smoothing) was performed on the PS values using R package stlplus with the following settings: n.p = 4, s.window = 5, t.degree = 1, s.degree = 1^77^. Molecular formula assignment was performed in DOMAssignR with standard settings and the following elements being considered: CHON_0-4_S_0-1_. The m/z values with successful sum formula assignment accounted for 83 ± 2% (mean ± standard deviation) of the total intensity of the respective DI-HR-MS spectra.

Effects of potential instrument drift due to the long measurement series were checked by repeatedly running an in-house groundwater DOM reference together with the samples and performing the same PS value calculations as described above. Figure 2b shows that there was no significant drift over the measurement sequences.

Metabolic pathway prediction from DOM DI-HR-MS data was carried out using the KEGGREST package^78^. Pathway predictions were constrained by a dereplicated, quality-controlled data set of 1224 MAGs obtained from the same well(s) in January 2019^54^. Predictions of metabolic pathways from DOM were filtered to match predictions from metagenomes of the same respective well. Detailed descriptions of the metabolic pathway prediction workflow and quality control are available in Supplementary Figs. 6,  7. Linking DOM and metagenomes provided insights beyond those achieved with traditional methods. More than forty distinct metabolic pathways were jointly predicted by DI-HR-MS and metagenomics data. In a previous investigation of DOM at the Hainich CZE, we evaluated four compound classes based on the Van Krevelen Diagram: polyphenols, highly unsaturated substances, unsaturated aliphatics, and peptide-like substances^34^. These structural features not only lacked a precise functional description but have since been shown to loosely overlap with their respective regions in the Van Krevelen Diagram^79^. Here, more than 70% of sum formulas could be unambiguously linked to specific metabolic pathways, largely due to the requirement for matching predictions based on metagenomes.

### Radiocarbon isotopes

SPE-DOM samples were dried in tin capsules, combusted to CO_2_ in an elemental analyzer, and graphitized. ^14^C measurements were carried out on a MICADAS accelerator mass spectrometer system (Ionplus, Dietikon, Switzerland). A detailed description of sample preparation and measurement recording can be found in ref. ^80^. In a previous investigation, it was found that our SPE procedure does not introduce a significant bias to the ^14^C/^12^C ratios compared to the original material^81^.

### Metagenomes

We studied a large metagenomics data set of Hainich CZE groundwater samples (six wells) that had been sampled in January 2019 and sequenced as previously described^82^. Metagenomic assemblies of raw sequencing reads (contigs or scaffolds) were generated using a previously described bioinformatics pipeline^82^, and used to predict genes with Prodigal v2.6.3^83^. Protein FASTA sequences derived from predicted coding genes were queried for sequence similarity against the KEGG Orthology (KO) database using KofamScan^84^, and resulting KO identifiers were mapped to KEGG metabolic pathways. Normalized abundances of each KO gene were derived based on the number of raw reads mapped to each gene sequence in the respective metagenome. BBmap (v.38.96)^85^ was used to map metagenomics reads.

## Supplementary information

Supplementary Information

Transparent Peer Review file

## Data Availability

Raw metagenomic sequencing reads for the studied groundwater samples were deposited under ENA project accession PRJEB36505, and respective assemblies were deposited under ENA project accession PRJEB36523. Raw DOM HR-MS data were deposited under 10.17617/3.WRNNZH.

## Code availability

Code for DOM raw data processing and statistical evaluations in R and a workspace image of processed data, including supplementary stable isotope and radiocarbon data, were deposited under 10.17617/3.WRNNZH. The code was tested in R version 4.4.0.
