# Supplementary material for: Hydroclimatic extremes threaten groundwater quality and stability
Source: Nat Commun. 2025 Jan 16;16:720. doi: 10.1038/s41467-025-55890-2 (PMC11739494; doi:10.1038/s41467-025-55890-2)
Supplement: Supplementary file 1 — Supplementary Information [file 41467_2025_55890_MOESM1_ESM.pdf]

Supplementary Information

Hydroclimatic extremes threaten groundwater quality and stability

Simon A. Schroeter[1]*, Alice May Orme[1], Katharina Lehmann[2], Robert Lehmann[2], Narendrakumar M. Chaudhari[3,4], Kirsten Küsel[3,4,5], He Wang[3], Anke Hildebrandt[4,6,7], Kai Uwe Totsche[2,5], Susan Trumbore[1], and Gerd Gleixner[1]

[1] Department of Biogeochemical Processes, Max Planck Institute for Biogeochemistry, Jena, Germany
[2] Department of Hydrogeology, Institute of Geosciences, Friedrich Schiller University, Jena, Germany
[3] Aquatic Geomicrobiology, Institute of Biodiversity, Friedrich Schiller University, Jena, Germany
[4] German Centre for Integrative Biodiversity Research (iDiv) Halle-Jena-Leipzig, Leipzig, Germany
[5] Balance of the Microverse, Cluster of Excellence, Friedrich Schiller University, Jena, Germany
[6] Department Computational Hydrosystems, Helmholtz-Centre for Environmental Science – UFZ, Leipzig, Germany
[7] Terrestrial Ecohydrology, Institute of Geosciences, Friedrich Schiller University, Jena
* Corresponding author: simon.schroeter@bgc-jena.mpg.de

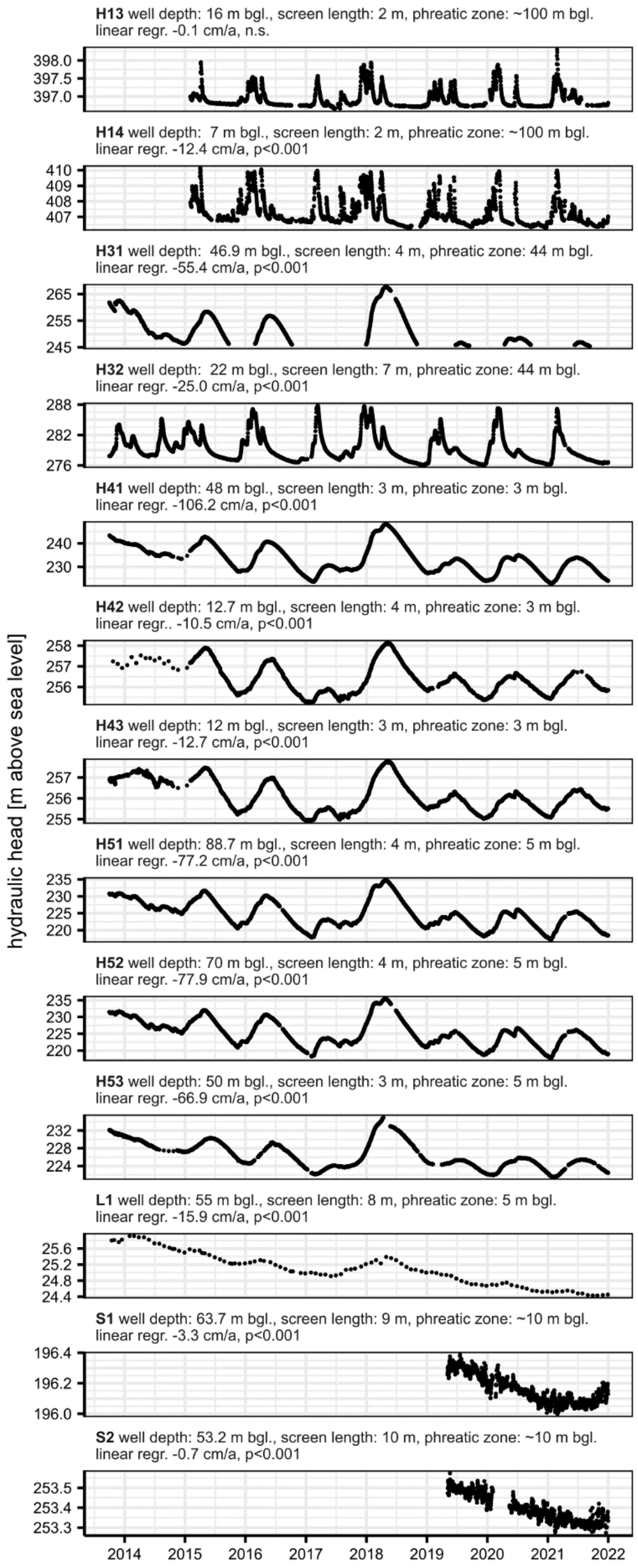

Supplementary Figure 1: **Groundwater hydraulic heads.** Well depths and depths to the phreatic zone are given in meters below ground level (m bgl.). Significant groundwater losses were observed in 12 out of 13 wells. Wells access aquifers in limestone-mudstone alternations at the Hainich CZE (well IDs starting with letter H), in glacial sands and gravels at the Forschungsstation Linde (well L1), and in sandstone-siltstone alternations at the SESO (wells S1, S2).

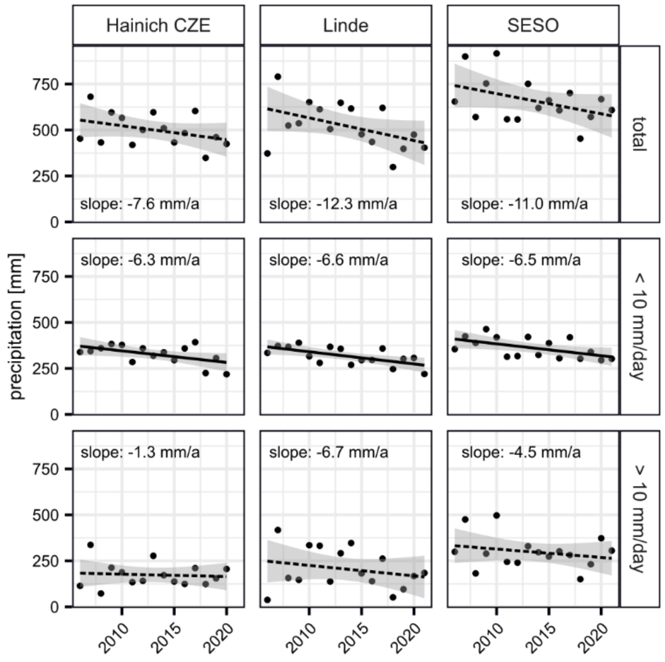

Supplementary Figure 2: **Yearly precipitation time series for the period 2006-2021.** All sites show decreases in yearly total precipitation. Decreases in heavy precipitation (>10 mm/day) are less than lighter precipitation losses at the Hainich CZE and SESO. At the Linde site, no differences between light and heavy precipitation losses are observed. Daily precipitation data was downloaded from Deutscher Wetterdienst (DWD) at https://opendata.dwd.de/climate_environment/CDC/observations_germany/climate/daily/kl/historical for the closest DWD weather stations to our groundwater investigation sites (stations #6305, #6265, and #3289 for Hainich CZE, Linde, and SESO, respectively). The definition of >10mm/day as heavy precipitation is based on the definition by the European Climate Assessment & Data Project (www.ecad.eu).

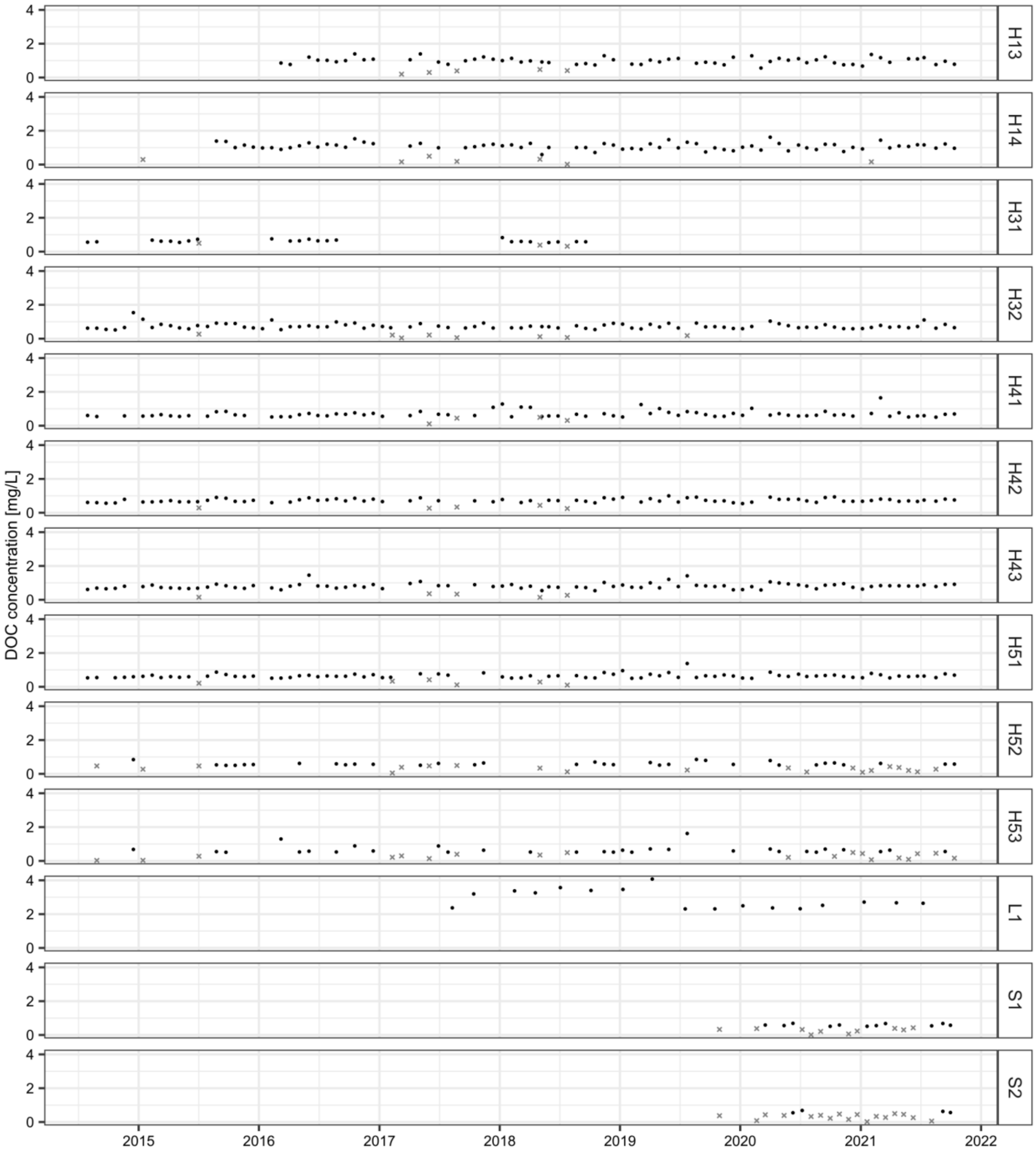

Supplementary Figure 3: **Time series of dissolved organic carbon (DOC) concentration in groundwater (n=777).** Measurement results below the limit of quantification of 0.5 mg/L are indicated as grey Xs and their plotted values are random between zero and 0.5 mg/L based on uniform distribution (R function *runif(max=0.5))*. Linear regressions per well yielded R^2 values ranging from $3.9*10^{-4}$ to 0.23. None of the slopes were significant (all Pr(>|t|) values in the slope outputs of R function *lm()* were greater than 0.05).

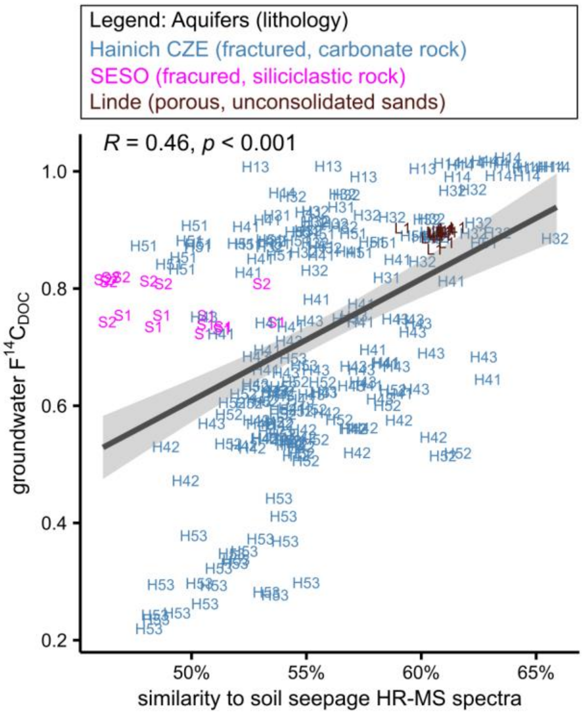

Supplementary Figure 4: **Pearson correlation between all similarity to soil seepage HR-MS spectra values reported in Fig. 2 and the fraction of modern carbon ($F^{14}C$) in groundwater DOM (n=238).** Resulting trend supports the validity of using DOM DI-HR-MS-based assessments to identify surface-derived impacts on natural groundwater quality. Very low $F^{14}C$ values in parts of the Hainich CZE wells (especially the anaerobic H53) reflect influence of carbon derived from host rocks and/or result of CO_2 fixation.

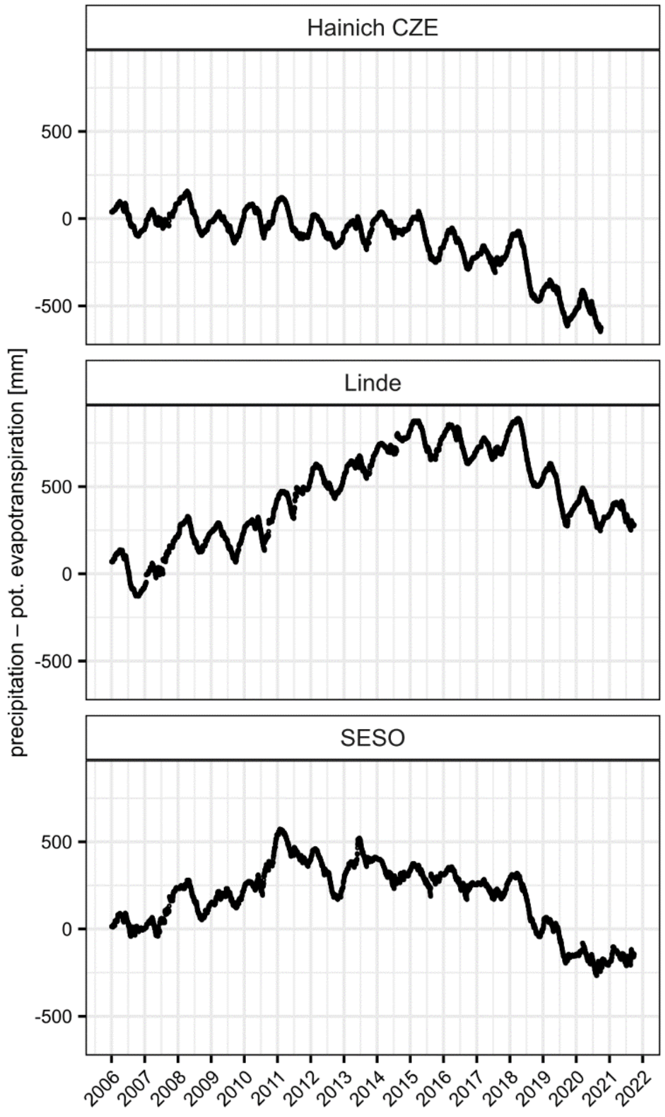

Supplementary Figure 5: **Cumulative water balance for the period 2006-2021.** The Europe-wide extreme drought year of 2018 shows as the largest annual water deficit in all investigated sites. Potential evapotranspiration was calculated using the Hargreaves-Samani-equation[66] (equation 8). Daily precipitation, as well as minimum, maximum and mean temperature data were downloaded from Deutscher Wetterdienst (DWD) at https://opendata.dwd.de/climate_environment/CDC/observations_germany/climate/daily/kl/historical for the closest DWD weather stations to our groundwater investigation sites (Hainich CZE station #6305, Linde station #6265, SESO station #3289).

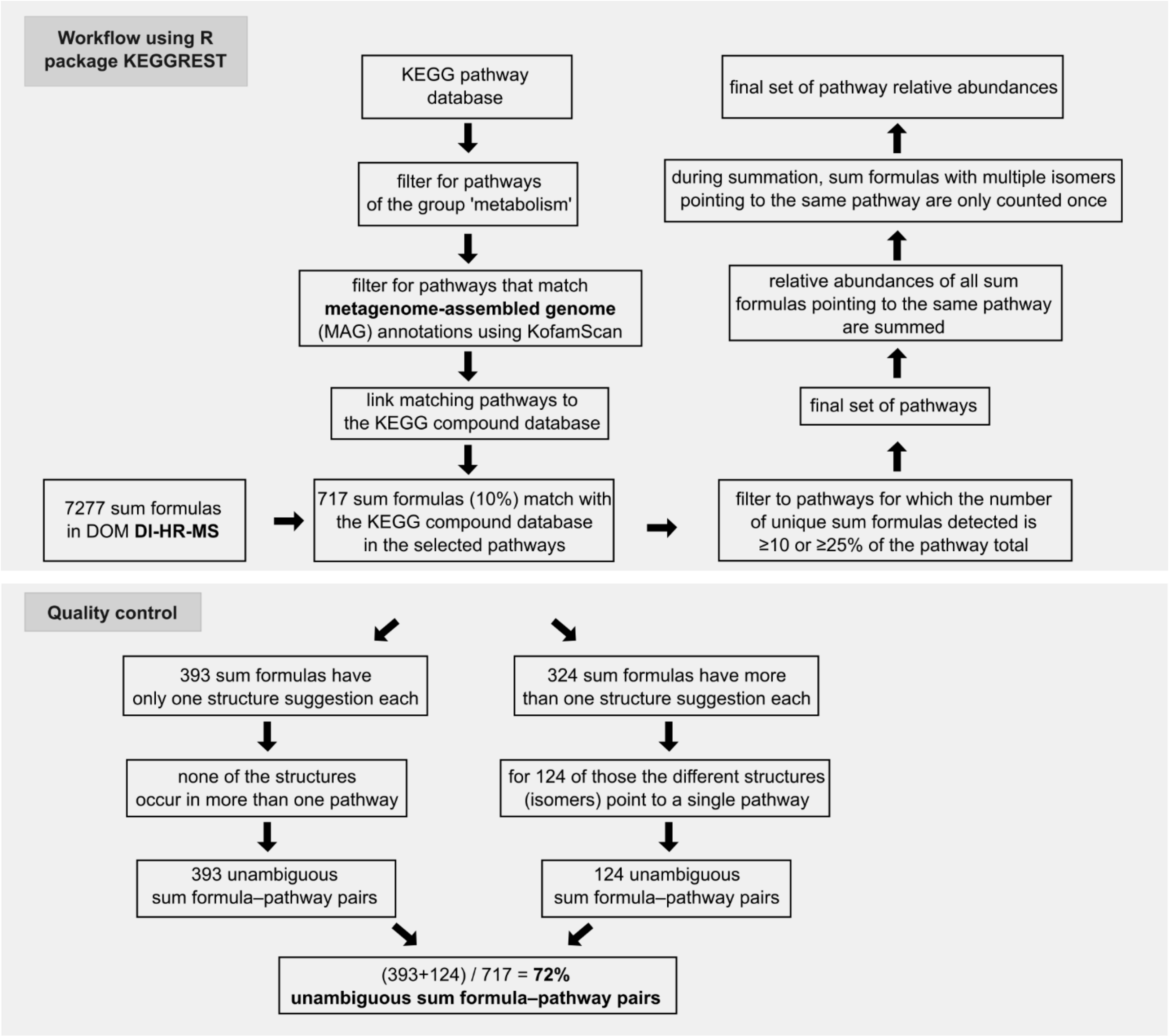

Supplementary Figure 6: **Overview of the workflow and quality control during the prediction of metabolic pathways via the KEGG database from DOM DI-HR-MS (n=254) and groundwater metagenomes (n=32).**

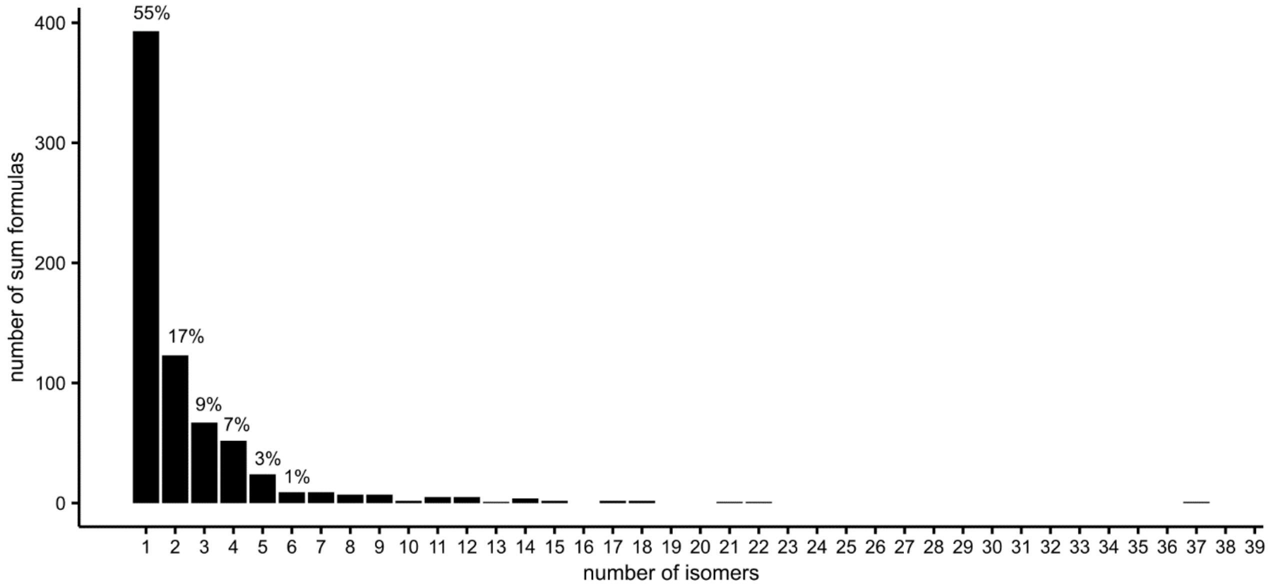

Supplementary Figure 7: **Number of hits in the KEGG compound database for each sum formula in DOM DI-HR-MS.** Non-matching sum formulas not shown. All sum formulas with single hits also only point to single pathways. For more than a third of the sum formulas with multiple matches in KEGG, the suggested isomers are part of the same metabolic pathway. Combined, these result in unambiguous pathway predictions for 72% of the sum formulas that matched with KEGG. Detailed descriptions of the workflow are available in Supplementary Figure 6.

Supplementary Table 1: **Year-on-year changeover in similarities between groundwater and soil seepage DOM HR-MS spectra.** Values represent the slopes of linear regressions of the percentage similarity values presented in Figure 2, split into before and after July 2018, the height of the extreme summer drought in Europe. Slopes of the linear regressions are given in percentage points per year (pp/a). Missing values (NA) indicate insufficient data during the respective period.

site	well	June 2014 to June 2018 [pp/a]	July 2018 to October 2021 [pp/a]
Hainich CZE	H13	NA	NA
	H14	NA	1.9
	H31	0.17	NA
	H32	0.886	0.994
	H41	-0.191	0.513
	H42	0.174	1.11
	H43	1.05	1.26
	H51	0.413	0.745
	H52	0.466	0.902
	H53	1.67	-0.504
Linde	L1	-1.12	0.148
SESO	S1	NA	0.916
	S2	NA	0.25
