# Supplementary material for: Hydroclimatic extremes threaten groundwater quality and stability
Source: Nat Commun. 2025 Jan 16;16:720. doi: 10.1038/s41467-025-55890-2 (PMC11739494; doi:10.1038/s41467-025-55890-2)
Supplement: Supplementary file 2 — Transparent Peer Review file [file 41467_2025_55890_MOESM2_ESM.pdf]

nature portfolio

Peer Review File

Hydroclimatic extremes threaten groundwater quality and stability

Corresponding Author: Dr Simon Schroeter

This file contains all reviewer reports in order by version, followed by all author rebuttals in order by version.

Version 0:

Reviewer comments:

Reviewer #1

(Remarks to the Author)
I co-reviewed this manuscript with one of the reviewers who provided the listed reports. This is part of the Nature Communications initiative to facilitate training in peer review and to provide appropriate recognition for Early Career Researchers who co-review manuscripts.

The authors report a unique dataset comprising HR-MS of groundwater organic carbon and associated parameters from Critical Zone Observatories, focusing on an 8-year record from a karst aquifer. This is a unique and valuable dataset that deserves publication. Our main comments relate to some areas where further methods clarification is requested, and several questions related to statistical analyses. The metagenome section is not reviewed as this is outside our expertise.

l. 18 – It would be good to specify 'more frequently' than what?

l. 43 Maybe consider referring to HR-MS techniques more generally, the authors use Oribitrap but the cited studies use a range of techniques including FT-ICR-MS.

l. 53 Only one site is 8-years, and the other two sites are short during and just one or two observations. This statement might mislead the reader.

l. 58. Somewhere in the manuscript, we would value some context for the groundwater samples e.g. redox state, salinity or EC. Something to help the reader understand if the groundwater environment is likely to support microbial degradation of DOM. At this location it would be helpful to know if all the sample sites are oxic (reading on, it seems at least one is not).

l. 69 Does this mean the maximum hydraulic head has declined every year, or is this the average (i.e., what is the -0.7 and -106 cm per year value referring to)?

l. 75 Do the authors know how much rainfall is needed to generate groundwater recharge? The time series in Supplemental Fig 1 for H13 and H14 show event type responses, so maybe this can be determined? We ask as I was not sure if events <10 mm would generate recharge, and whether the statements here were correct.

l. 77 –Fig 1 doesn't seem to show a declining trend in the maximum d18O values observed (max values still ~ between -8.25 to -8.75 ‰), it seems to be the minimum d18O values are declining. It would be good to discuss the reason for this. E.g., if there are less small-scale precipitation events impacting groundwater, wouldn't we expect to also see a decrease in the max d18O values?
l. 79 and Fig 1 - Is greater variability in the d18O through time the result of an increasing number of sampling points/bores being sampled? We suggest adding sample number of each year in Fig 1B. Fig 1B also appears to show a return to lower d18O variance in 2021 – what are the reasons for this?
l. 83 Is the increased variability in d18O occurring any particular season, or is this occurring in all seasons?

Could the authors provide some more clarification on exactly what the Bray-Curtis dissimilarity was calculated on? An extra line in the methods would help, was it on all the m/z for each sample?

The authors remove the heterogeneity of the soil HR-MS spectra by taking a single value. It would be good to quantify and visualise the distribution of soil HR-MS so that the reader can get some context of the heterogeneity. We were wanting to understand if was greater or less than than the long term trend shown in Figure 2.
Fig 1a We found this hard to read the data and the Linde sites seemed obscured.

Fig 1b. We need convincing of this analysis. When calculating the intra-annual variability – was this on a calendar year? Should this be determined on the hydrological year? Also, the data is not normally distributed and is also temporally autocorrelated. How is the r value calculated, is it Spearmans' rho or Kendall's Tau – is it? In any case, the increase in variance over time is less than the precision of the analyses, so I would be very cautious with this interpretation.
Also, please check for typos in the figure e.g. Hinch vs Hainich, 'sans' in caption

l. 100 - It would be useful for a reader if you could include a sentence explaining the mechanisms causing preferential flow paths to be more impacted than the unconsolidated sands.

l. 105 and Supp Fig 3 – It is suggested that the authors provide the range of R2 and P values. What is the mechanism for a lack of DOC increase? Is this reflective of greater dilution in extreme rainfall events that are leading to the transport of surface molecules into groundwater?

l. 106 and Figure S3. The DOC data is a crucial part of the argument e.g. it is stated that it had remained constant. A statistical analysis would be helpful to confirm the gradient is indistinguishable from zero, noting that these are low DOC values, the LOD is 0.5 mg/l and many values will need to be replaced by random numbers below the LOD. What is the precision of an individual analysis?

l. 109 and Suppl Fig 4. The authors write that this is a 'strong' relationship. It wasn't clear to us what the r value is calculated on, is it all the data from all sites? We are told here that H53 is anaerobic, and this bore clusters separately with older DOM – does this one site influence the r value. What is the r value for the oxic sites alone? The question we had was - is there no relationship between age and PS for all the oxic sites.

l. 109 – We'd be interested to see if groundwater 14C DOC age also shows decreasing DOC age over time in the bores? The authors should provide the raw 14C DOC and d18O datasets in the Supplementary Material as it was not possible to examine the isotope data in detail.

l. 105 – The authors write that this is a 'significant' increase. How is this significance determined?

l. 114 onwards – the authors argue that there is a change in composition after 2018, based on changeover values on linear regressions (Suppl Table 1). Could the authors clarify that this is this an analysis based on the data shown in Fig 2c? How are the changes in gradient tested statistically? Are they bigger than the uncertainty on the gradient?

l.123 – It is noted here that max hydraulic heads were in 2017, although it looks like the highest hydraulic heads may have been early 2018 in the Hainich CZE on Supp Fig 1

l. 245 Methods – what bottles were used to store the stable water isotope, 14C and DOC/DOM samples?

L323 –Is *100 missing from the formula for percent similarity? I.e: $1-(\textit{Bray Curtis dissimilarity})$ *100?

The authors should specify the software/packages used to create all figures in each caption.

Andy Baker (UNSW) and Liza McDonough (ANSTO)

(Remarks on code availability)

Reviewer #2

(Remarks to the Author)
I co-reviewed this manuscript with one of the reviewers who provided the listed reports. This is part of the Nature Communications initiative to facilitate training in peer review and to provide appropriate recognition for Early Career Researchers who co-review manuscripts.

(Remarks on code availability)
The code is available in the supplementary material and Github. It all looks to be complete however I wasn't able to install package "MFAssignR" to run the code for the raw data import.

Reviewer #3

(Remarks to the Author)
This paper discusses the relationship between groundwater levels, the concentration and composition of dissolved organic matter (DOM) in groundwater, and the source of this DOM in Europe between 2014-2021. The paper highlights the changes in observed groundwater levels and molecular DOM composition in response to hydroclimatic extremes, comparing these

changes to the composition of DOM from surface (soil-seepage) derived sources. The authors conclude that hydroclimatic extreme events (i.e., periods of intense rainfall) promote the rapid movement of water and DOM between shallow surficial seepage and deeper groundwater aquifers at three Critical Zone sites, and that aquifer DOM is more similar to soil-seepage derived DOM following these hydroclimatic extreme events than under normal conditions. The authors conclude that this is because surface derived DOM moved through the aquifer unaltered by microbial processes that would otherwise degrade this DOM during its transport. In turn, the movement of unaltered DOM poses a concern for the safety of the aquifer drinking water resource, promoting aquifer redox conditions that may further contaminate the aquifer following hydroclimatic events. The authors discuss that the occurrence of these events is increasing, which in response, may exacerbate the deterioration of groundwater drinking water quality. The authors highlight the usefulness of DOM DI-HR-MS as a tool for detecting changes in groundwater deterioration and linking these changes to their source contributions. The authors also employ the KEGG database to predict relative abundances of metabolites that can be used to infer the metabolic functions of the groundwater microbiome. The study analyzed an impressive number of groundwater and soil seepage samples from three distinct critical zone research sites, including for DOC concentrations, stable hydrogen and oxygen isotope composition of the waters, radiocarbon of DOC, and DI-HR-MS. The study also predicted relative abundances of functional metabolites and microbial gene products recorded in 32 groundwater metagenomes.

Overall, I find this paper could be a valuable contribution to the scientific community. The paper is appropriate for Nature Communications and would be of interest to its broad reader base. Given the study's use of data across space, time, and different types of measurements that each can provide source and process-based, mechanistic, understanding, I found that the authors need to expand the background, context, and elaborate on their findings in several areas of the paper. The authors share a concise story, but perhaps too concise given the complexity of the study. Nature Communications allows a generous word limit (5,000) that the authors should consider taking advantage of. By my estimate, the main body (not including Abstract, Methods, References, and Figure legends) is less than 1,000 words.

In my section and line by line comments below, I provide suggestions for the areas the authors could expand upon, with the goal of setting up a stronger background in the Introduction and elaborating on process-based and mechanistic linkages between the results and conclusions in the Results and Discussion.

Introduction

In Lines 33:35 the authors state that the "availability of valuable clean groundwater is threatened by increasingly extreme hydroclimatic conditions that alter the functioning of natural recharging processes" but they do not explain what an extreme hydroclimatic condition is, why type they are referring to, nor how their increasing occurrence alters the function of natural recharging processes that may threaten groundwater quality. It's then difficult for the reader to interpret the study's main hypothesis in Line 38:40. Likewise, here the connection to the mechanism is not being described. What are the natural filtering and transformative processes that render groundwater clean and safe to drink? Examples that relate to those processes that the results of this study inform would be helpful.

Lines 43:46 – The authors need to first explain what DI-HR-MS measures before going into how the measurement has helped scientists understand DOM's role in mediating key biogeochemical processes. Similar to my comment above, including specific examples would be helpful here. How has DI-HR-MS helped scientists understand sorption of surface-derived substances to minerals and their active transformation. What kind of substances and what are they transformed into?

Lines 46:48 – Why have studies suggested DOM DI-HR-MS data can be used as a proxy for the aquifer properties mentioned here? Please elaborate.

Lines 48:49 – There is little prior work mentioned that gives context to why long-term and large-scale efforts aimed at exploiting DOM DI-HR-MS data to infer groundwater quality under variable and extreme conditions is specifically needed (i.e., as opposed to other types of DOM measurements).

Lines 58:64 – This space has been used to give the reader details that are repeated in the Methods. It would be valuable to use this space to elaborate on the study's approach to addressing its research question/hypothesis, the purpose behind the measurements/analysis, and how they are being used in concert.

Results and Discussion

Lines 77:84 – Please elaborate how the patterns or changes in stable water isotope results specifically indicate the conclusions stated in this section.

Lines 91:97 – What additional context or reference to other studies can be used to justify that the 10%, 5% and 1% DOM composition similarity is a significant change for the timeframe evaluated, 2014-2021? DI-HR-MS provides a compositional analysis but there is no mention of the types of compounds that became more similar or different in groundwater in comparison to their abundance in surficial soil-seepage over the study's timeframe. With this much valuable information on source and biogeochemical signatures, how come the authors didn't consider applying a mixing model to estimate proportional contributions of soil-seepage DOM to the groundwater DOM pool over time?

Lines 97:99 – Likewise, what specific changes in the DOM molecular compounds, where, and at what times provide evidence of an intensifying soil-to-groundwater connection, coupled to a reduction in efficiency of natural filtrations and purifications that occur during percolation from the vadose zone into the aquifers?

Lines 100:102 – Please elaborate on the results that led the authors to reach this conclusion.

Lines 104: 107 – What process-based understanding can the authors provide to explain why DOC concentration did not show significant changes during the observation period, and what are the implication of this finding?

Lines 107-113 – How does the 14C results reinforce the validity-based DOM assessments. Please be explicit and point to the patterns observed with reference to other studies that indicate this is true. Could modern sources of C be introduced from a different pathway?

Lines 126:129 – Which substances? And how does this provide evidence that this DOM had not been processed microbially and had evaded Critical Zone filtration and purification processes?

In the "Implications for groundwater ecosystems" section, are there additional linkages that can be made between microbially-mediated metabolic pathways and specific changes in DOM compound classes/composition before and following extreme hydroclimatic events that shed light onto the potential for contaminant release?

(Remarks on code availability)
The authors provide the DOM processing code using R computing software, which appears it can be easily reproduced.

Reviewer #4

(Remarks to the Author)
Schroeter et al. present a comprehensive investigation of DOM composition, source, and dynamics in natural groundwater in relation to precipitation patterns in central Europe over 8 years. Most of the data is from one particular CZO in Germany, but two other central European CZOs are also included, although with much fewer observational data points.

The authors make a case that DI-HR-MS analyses of DOM can serve as a proxy for groundwater quality degradation, based on its ability to detect compounds related to microbial metabolic pathways involved in xenobiotics' degradation and biosynthesis of toxins and anti-biotics in natural groundwater. They also show how hydroclimatic extremes may compromise the filtration and purification effect that soils typically provide for precipitation water percolating through the soil, by promoting quick transport to groundwater, presumably through preferential flow, and thereby increasing the presence of surface-derived organic carbon (including xenobiotics) in groundwater, potentially leading to groundwater quality degradation.

The amount of observational data included in this study is impressive and I find the approach generally sound and the results interesting. However, I think that the brevity of the text has somewhat limited the authors to deliver clear and substantiated support from the data to some of their statements and the message being conveyed is somewhat convoluted to me. For example, it took me several thorough readings of the manuscript to understand that the only metabolic pathways that were actually considered from the KEGGS database and linked to the DI-HR-MS data were those concerning xenobiotics and biosynthesized toxins and anitbiotics. There is also a lack of detail explaining how some of the calculations were made and the motivation for those with commentary on potential limitations, assumptions etc associated with the statistical and other data processing/comparisons/interpretations being conducted. I have tried to pinpoint some of these aspects in my detailed comments below.

I think this study is well conducted and the results should be of interest to the scientific community, but I cannot be sure, because the text is not providing enough detail and focus to fully assess the quality of the study and relevance of results. This, together with the somewhat narrow scope of "only" including xenobiotics and biosynthesized toxins and antibiotics (which definitely should be made clear very early on to avoid misunderstandings of what "metabolic pathways" are considered in this study) puts this slightly outside of what I would consider suitable for Nature Communications. I think it may fit better in a slightly less broad scientific journal. However, if edits were made to address these concerns, it may well be that this manuscript could be suitable for Nature Communications, especially if a bit more effort was put towards clarifying how the broader statements being made are supported by the data and also put into a context of the importance of xenobiotics and biosynthesized toxins and antibiotics for groundwater quality in more concrete terms.

Detailed comments

L26 Suggest changing "naturally occurring xenobiotic substances" to "xenobiotic substances occurring in natural groundwater" as xenobiotics by definition are not "natural". Also, the biosynthesized antibiotics and toxins should not be considered "xenobiotics", and "xenobiotics" cannot be biosynthesized (then they would not be "xenobiotic"). This is also not completely clear in other parts of the manuscript (e.g., L217 in Fig. 3 caption)

L54-55 Suggest refraining from assertively stating that the study is "the most comprehensive" unless you have actual concrete proof of it – in which case this should be cited or at least supported by how you arrived at this conclusion. Better to state "one of the most comprehensive" or similar.

L114 (and supplementary Table 1, see comment below) It is not clear to me how the year-on-year changeover is calculated.

L120-121 “anthropogenic warming” sounds like it is intentional warming (e.g., as part of an experiment), suggest changing to “anthropogenic-induced global warming”

L142 (or somewhere else in this section) Suggest specifying that the targeted metabolic pathways were related to “xenobiotic degradation” and “natural toxin and antibiotic biosynthesis”, assuming that this is indeed the case based on results presented.

Fig. 1 The Hainich (blue) text is blurry to the extent that it is difficult to look at the figure. I understand there are a lot of points, but would be good if text could be sharper.

Fig. 2 I find “decomposition” to be confusing in the figure title – what is meant by this term here? Also, the sentence on L201-202 is not clear and the explanation in the methods section (L323-325) does not fully clarify what samples were compared to each other (all sites at all times? By site? How were the temporal trends assessed in that case?). Please provide more specifics to help clarify what was done (can go in Supplementary Information if needed)

Fig. 3 Suggest referring to Supplemental Figure 6 that explains the workflow for connecting DI-HR-MS data to KEGG. It is also not clear to me how the 254 DOM samples connect to the 32 genome samples. Are all DOM data for a specific sampling location (independent of time point) compared to a single groundwater genome data point? And were the groundwater genome data generated from any of the samples used for DOM analyses or were these from previous samples that had been collected and sequenced at an earlier stage (this is somewhat implied on L347)? The current writing in both the manuscript and the methods section are ambiguous. It is important to understand the relationship between the samples that were used for the two types of analyses in order to evaluate the relevance of data correlations.

Throughout Methods section – For all instruments, please add models and manufacturer locations

L272-273 Suggest adding a bit more detail about the lysimeters, although the information is provided in the cited publications. For example, although the lysimeters installed at these sites are indeed the “original” kind – i.e. porous plates placed underneath a soil-filled cylinder, recent use of the term has been applied to other types of suction pore water samplers, so it would be prudent to specify the type used here. It would also be nice as a reader to get the dimensions, pore size and material of the porous plate, and collection depth(s) without needing to look up the citations. Also, for Hainich – if I understand correctly) lysimeters are installed at 2 depths – were samples collected from both or only at the topsoil/subsoil interface?

L 326 What is meant “by loess” here?

L329 Why was P not considered in the formula assignments?

Supplementary Information

Supplementary Figure 7 Very minor, but the percentage of unambiguous pathways predictions was 72% in Supplementary Figure 6 (which matches the numbers shown in that figure), so just wondering why it would be stated as 73% in the caption here.

Supplementary Table 1 I am confused by what is meant by “Year-on-year changeover” when in the next sentence it is stated that linear regressions were calculated for data before and after July 2018 (i.e. multiple years for each regression). It makes sense to divide the time series this way based on the 2018 drought induced shift, but I am struggling to understand how the year-on-year changeover similarities is calculated. Also, it is not clear to me what the numbers presented in the table actually represent. What does pp stand for in the units used?

(Remarks on code availability)
I didn't see any code included i the supplementary materials

Version 1:

Reviewer comments:

Reviewer #1

(Remarks to the Author)
This is again a co-review by Andy Baker (UNSW) and Liza McDonough (ANSTO).

We thank the authors for their thorough response to the reviewers, and the updated code.

We (that is, Andy) have only one question remaining which relates to Figure 1b. Thank you for confirming the normality of this data, and that the analysis is on calendar years. We suggest the latter information is included in the figure caption? However, I am still not convinced that the statistic provided is robust. For example, I couldn't see that the question of temporal autocorrelation was addressed. If the individual time series are temporally autocorrelated, this will affect the significance of the correlation coefficient. Also, the y-error is 0.1 per mille on each analysis, but is this included in the

regression analysis? My original concern remains - the analytical uncertainty in each data point is greater than the trend in this data, the r value is not that strong, and the data from all the bores has been combined in order to produce this statistic. This reviewer (Andy) would have greater confidence in this analysis if it was demonstrated to reproducible e.g. on a repeat analysis where hydrological years were used, and/or a repeat analysis where the y-error was included and/or Kendall's tau was used.

(Remarks on code availability)
Thank you for the revised code which now seems to work and contains the 14C and d18O data. I noticed that I had to add Library(broom) to the top of the code in order to run the piece of code titled #LINEAR REGRESSIONS OF SIMILARITIES TO SOIL SEEPAGE DOM. It is suggested that this library gets added to the supplementary code prior to final upload.

Reviewer #2

(Remarks to the Author)
I co-reviewed this manuscript with one of the reviewers who provided the listed reports. This is part of the Nature Communications initiative to facilitate training in peer review and to provide appropriate recognition for Early Career Researchers who co-review manuscripts.

(Remarks on code availability)
See comments from co-reviewer.

Reviewer #3

(Remarks to the Author)
The authors seemed to have thoughtfully considered the reviewer suggestions and made subsequent changes to address the concerns raised in the reviewer comments. I find that the changes made improve the paper overall.

In their response letter, the authors explain why they chose not to use the DI-HR-MS DOM composition data to infer types of compound classes via the van Krevelen Diagram, and in turn use similarities, differences, and/or changes in the relative abundances of these compound classes in the study's analysis, as most studies do. The authors state that the study's multi-omics prediction approach, linking DOM composition to microbial community functions, far surpass this type of compound predictions both in molecular detail and prediction accuracy, and thus provide an improved alternative to compound class predictions from DI-HR-MS DOM data. Since compound classification of DI-HR-MS DOM composition data is a commonly applied technique, the argument against inferring compound classes via the van Krevelen Diagram and the reasons for implementing the alternative approach as done in the study and in the future should be discussed in greater detail in the paper.

(Remarks on code availability)

Reviewer #4

(Remarks to the Author)
The manuscript has been greatly improved and my concerns have been met. I apologize to the authors for missing some additional material in the first round.

(Remarks on code availability)

Version 2:

Reviewer comments:

Reviewer #1

(Remarks to the Author)
No further comments. Thank you for addressing our questions

(Remarks on code availability)

Reviewer #2

(Remarks to the Author)
I co-reviewed this manuscript with one of the reviewers who provided the listed reports. This is part of the Nature Communications initiative to facilitate training in peer review and to provide appropriate recognition for Early Career Researchers who co-review manuscripts.

(Remarks on code availability)

Reviewer #3

(Remarks to the Author)
The authors have adequately addressed my comment, thank you.

(Remarks on code availability)

Open Access This Peer Review File is licensed under a Creative Commons Attribution 4.0 International License, which permits use, sharing, adaptation, distribution and reproduction in any medium or format, as long as you give appropriate credit to the original author(s) and the source, provide a link to the Creative Commons license, and indicate if changes were made.
In cases where reviewers are anonymous, credit should be given to 'Anonymous Referee' and the source.
The images or other third party material in this Peer Review File are included in the article's Creative Commons license, unless indicated otherwise in a credit line to the material. If material is not included in the article's Creative Commons license and your intended use is not permitted by statutory regulation or exceeds the permitted use, you will need to obtain permission directly from the copyright holder.
To view a copy of this license, visit https://creativecommons.org/licenses/by/4.0/

REVIEWER COMMENTS

Reviewer #1 (Remarks to the Author):

I co-reviewed this manuscript with one of the reviewers who provided the listed reports. This is part of the Nature Communications initiative to facilitate training in peer review and to provide appropriate recognition for Early Career Researchers who co-review manuscripts.

The authors report a unique dataset comprising HR-MS of groundwater organic carbon and associated parameters from Critical Zone Observatories, focusing on an 8-year record from a karst aquifer. This is a unique and valuable dataset that deserves publication. Our main comments relate to some areas where further methods clarification is requested, and several questions related to statistical analyses. The metagenome section is not reviewed as this is outside our expertise.

A: Dear Liza and Andy, I am very happy that the editor follwed my suggestion to send the paper to you. Thank you for the helpful suggestions!

l. 18 – It would be good to specify 'more frequently' than what?

A: As there is no defined reference timeframe, this sentence was rephrased and now reads: „Heavy precipitation, drought, and other hydroclimatic extremes occur more frequently than in the past climate reference period (1961-1990).“

l. 43 Maybe consider referring to HR-MS techniques more generally, the authors use Oribitrap but the cited studies use a range of techniques including FT-ICR-MS.

A: In accordance with comments by the other reviewers, we added more general methods information here. The section now reads: "DI-HR-MS does not rely on chromatographic separation, but on the great mass resolution provided by FT-ICR-MS or, here, Orbitrap analyzers. Resulting data reveal relative abundances of thousands of distinct molecular entities. Relative abundance patterns of the same sample type can differ between instrument types and change with method parameters31. Knowing this, we used a single method and measured repeated standards to be able to demonstrate the scalability of DI-HR-MS analyses for long-term and large-scale efforts aimed at exploiting DOM molecular composition data to infer groundwater quality under variable and extreme conditions."

l. 53 Only one site is 8-years, and the other two sites are short during and just one or two observations. This statement might mislead the reader.

A: Agreed, the respective sentence now reads: „Considering time series of up to eight years…“

l. 58. Somewhere in the manuscript, we would value some context for the groundwater samples e.g. redox state, salinity or EC. Something to help the reader understand if the groundwater environment is likely to support microbial degradation of DOM. At this location it would be helpful to know if all the sample sites are oxic (reading on, it seems at least one is not).

A: As suggested, we added more details on the hydrochemistry of the groundwater to the site description in the method section. The following was added: „Aquifer compartments at the Hainich CZE differ strongly in oxygen availability. Wells H13, H14, H31, H32, H41, and H51 are oxic, H42 and H43, H52 and H53 are anoxic (<0.1 mg/L dissolved oxygen). Redox potentials range around 400 mV for wells H13, H14, H31, H32, H41, and H51, and around 200 mV for wells H42, H43, H52, and H53. (…) Groundwater accessed at SESO is oxic. (…) Groundwater accessed at the Forschungsstation Linde is oxic.

l. 69 Does this mean the maximum hydraulic head has declined every year, or is this the average (i.e., what is the -0.7 and -106 cm per year value referring to)?

A: Supplementary Figure 1 specifies that the values refer to linear regressions, so it's the average decline per year.

l. 75 Do the authors know how much rainfall is needed to generate groundwater recharge? The time series in Supplemental Fig 1 for H13 and H14 show event type responses, so maybe this can be determined? We ask as I was not sure if events <10 mm would generate recharge, and whether the statements here were correct.

A: The definition of 10mm per day as the threshold value between light and heavy precipitation is based on the definition by the European Climate Assessment & Data Project (www.ecad.eu). This is now mentioned in the caption of Supplementary Figure 2. A general threshold value for the generation of recharge cannot be provided for (soil-covered) landscapes with variable soil types and thicknesses, particularly because groundwater recharge depends on the preceding conditions of rainfall events (especially soil moisture among others). This differs significantly, for example, between summer, with high evapotranspiration, and the winter months, which have higher soil moisture levels, during which even small amounts of precipitation can lead to deep percolation. In winter, recharge is obvious from level responses to most extreme rain events (>21 mm/d), whereas for summer extremes (>30 mm), response can be absent. See Lehmann and Totsche (2020) for an analysis water level time series and further insights into the hydrogeological funtioning of the Hainich site.

l. 77 –Fig 1 doesn't seem to show a declining trend in the maximum d18O values observed (max values still ~ between -8.25 to -8.75 ‰), it seems to be the minimum d18O values are declining. It would be good to discuss the reason for this. E.g., if there are less small-scale precipitation events impacting groundwater, wouldn't we expect to also see a decrease in the max d18O values?

A: We're not quite clear on the expectation of declining maximum d18O values upon heavier rainfall. Do you mean because of the „amount effect" in stable isptope fractionation during precipitation? Not sure if we can expect to trace this effect into a pool as large as groundwater, where a lot of mixing of water from different sources (spatially and temporally) takes place. Observerd increasing maxima and decreasing minima rather suggest general differences in infiltration/mixing behaviour, which precipitation intensity and soil dryness are major drivers of.

l. 79 and Fig 1 - Is greater variability in the d18O through time the result of an increasing number of sampling points/bores being sampled? We suggest adding sample number of each year in Fig 1B. Fig 1B also appears to show a return to lower d18O variance in 2021 – what are the reasons for this?

A: Changes in variance cannot realistically increase with the number of observations because here the unbiased sample variance is used which features division by (n-1). Its equation is $var = \sum(x-\bar{x})^2/(n-1)$ *with* $(x-\bar{x})$ *as the deviation from the mean and n as the number of observations. Regarding the little bit lower variance in 2021, we're not sure of the reasons yet. It might potentially be an early indication of the (positive) impacts of a wetter climate in recent years in Germany. As 2021 is the endpoint of the current time series, we don't really dare to speculate though.*

l. 83 Is the increased variability in d18O occurring any particular season, or is this occurring in all seasons?

A: There is no strong effect within individual seasons. In our opinion, the driving force behind the increase in variance is heavy precipitation leading to a stronger influence of the difference in precipitation stable isotope signature between the seasons onto groundwater. Therefore, the effect is

visible only at the annual level.
Could the authors provide some more clarification on exactly what the Bray-Curtis dissimilarity was calculated on? An extra line in the methods would help, was it on all the m/z for each sample?

A: Correct, all m/z values for each sample were used. The respective sentence in the methods now reads: „The percentage similarity (PS) between groundwater and soil seepage HR-MS DOM spectra was calculated as $PS = 1 - (Bray\ Curtis\ dissimilarity)$ based on all m/z values above the S/N threshold."

The authors remove the heterogeneity of the soil HR-MS spectra by taking a single value. It would be good to quantify and visualise the distribution of soil HR-MS so that the reader can get some context of the heterogeneity. We were wanting to understand if was greater or less than than the long term trend shown in Figure 2.

A: Thank you for this very helpful suggestion. We quantified soil seepage heterogeneity based on percentage similarity the same way we did for groundwater, but instead of averaging over soil seepage, we averaged over groundwater. The result is a similarity of soil seepage to groundwater of 44 ± 5% (mean ± sd). The heterogeneity of soil seepage DOM composition is thus by a factor of 2 lower than the long-term trend observed in groundwater. We added this result to the caption of Figure 2. The respective code to retrieve these values is „bray_seepage <- c(rowMeans(distanceMatrix) %>% mean, rowMeans(distanceMatrix) %>% sd)". We added this line of code to the supplement.

Fig 1a We found this hard to read the data and the Linde sites seemed obscured.
A: We agree that it was hard to disentangle individual wells from Figure 1a. We changed the figure to show points instead of text to improve the figure's focus. This way the key message, that the increase in variance shown in Figure 1b indeed corresponds to an increase in the spread between observed maximum and minimum d18O values, is now emphasized. The color scheme for the Linde site was adapted to improve visibility. In case there is a problem with resolution of the figure, all main figures are available as vector graphics on the review platform.

Fig 1b. We need convincing of this analysis. When calculating the intra-annual variability – was this on a calendar year? Should this be determined on the hydrological year? Also, the data is not normally distributed and is also temporally autocorrelated. How is the r value calculated, is it Spearmans' rho or Kendall's Tau – is it? In any case, the increase in variance over time is less than the precision of the analyses, so I would be very cautious with this interpretation.
Also, please check for typos in the figure e.g. Hinch vs Hainich, 'sans' in caption

A: Yes, the annual variability was calculated on calendar years. The R value is the coefficient to describe the linear regression between all variances from Hainich CZE. We provide below a Shapiro-Wilk normality test to show that the variances are indeed normally distributed. The variance being lower than the precision of the analyses is not a problem, because of how the variance is calculated. The equation $var = \sum(x - \bar{x})^2/(n-1)$ shows that the deviation from the mean is divided by the number of observations minus one. Only if the deviations from the mean were lower than the precision of the analyses would there be a problem.

Shapiro-Wilk normality test

data: var$variance

W = 0.83239, p-value = 4.342e-08

l. 100 - It would be useful for a reader if you could include a sentence explaining the mechanisms causing preferential flow paths to be more impacted than the unconsolidated sands.

A: This section was rephrased. It now reads: „Fracture-flow aquifer systems appeared markedly more vulnerable than groundwater in porous, unconsolidated sands, considering the rates of change being an order of magnitude lower at the Linde site compared to Hainich CZE and SESO (Supplementary Table 1). This highlights the important potential of preferential flow paths along fractures in the subsurface, allowing surface-derived substances to evade purification processes during rapid groundwater infiltration. The role of fractures in providing preferential flow paths is even more important for marine bedrock like those of the Hainich CZE because of their matrix tightness (low porosity and permeability)45."

l. 105 and Supp Fig 3 – It is suggested that the authors provide the range of R2 and P values. What is the mechanism for a lack of DOC increase? Is this reflective of greater dilution in extreme rainfall events that are leading to the transport of surface molecules into groundwater?

A: Seed also below. We added: "It is likely that the previously shown great bioavailability of surface-derived DOC in groundwater contributed to the stability of groundwater DOC. The change in DOM composition would thus be expected to be driven rather by microbial metabolites and surface-derived substances, which are not readily biodegradable and not individually highly abundant."

l. 106 and Figure S3. The DOC data is a crucial part of the argument e.g. it is stated that it had remained constant. A statistical analysis would be helpful to confirm the gradient is indistinguishable from zero, noting that these are low DOC values, the LOD is 0.5 mg/l and many values will need to be replaced by random numbers below the LOD. What is the precision of an individual analysis?

A: We thank the reviewers for this very important suggestion. We did as suggested and replaced all values below 0.5 mg/L by random numbers. The slopes of all linear regressions were not significant. The caption of Supplementary Figure 3 now reads: „Time series of dissolved organic carbon (DOC) concentration in groundwater (n=777). Measurement results below the limit of quantification of 0.5 mg/L are indicated as grey Xs and their plotted values are random between zero and 0.5 mg/L based on uniform distribution (R function runif(max=0.5)). Linear regressions per well yielded R2 values ranging from $3.9*10^{-4}$ to 0.23. None of the slopes were significant (all Pr(>|t|) values in the slope outputs of R function lm() were greater than 0.05)."

l. 109 and Suppl Fig 4. The authors write that this is a 'strong' relationship. It wasn't clear to us what the r value is calculated on, is it all the data from all sites? We are told here that H53 is anaerobic, and this bore clusters separately with older DOM – does this one site influence the r value. What is the r value for the oxic sites alone? The question we had was - is there no relationship between age and PS for all the oxic sites.

A: Yes, the correlation considered all available data. As suggested, we re-ran the correlation for oxic wells at the Hainich CZE site only. These are: "H13", "H14", "H31", "H32", "H41", and "H51". The correlation was still significant (p<0.001). The result of R function cor.test() is pasted below. We added the following to the manuscript text: „The correlation was not heavily driven by low F14C values in the anoxic wells H52 and H53 and was also significant (p < 0.001) when considering only oxic groundwater from the Hainich CZE."

Pearson's product-moment correlation

data: brayRadiocarbon %>% filter(wells %in% c("H13", "H14", "H31", "H32", "H41", "H51")) %>% pull(percentSimilarity) and brayRadiocarbon %>% filter(wells %in% c("H13", "H14", "H31", "H32", "H41", "H51")) %>% pull(F14C)

t = 3.659, df = 101, p-value = 0.0004046

alternative hypothesis: true correlation is not equal to 0

95 percent confidence interval:

0.1591269 0.5023793

sample estimates:

cor

0.3421161

l. 109 – We'd be interested to see if groundwater 14C DOC age also shows decreasing DOC age over time in the bores? The authors should provide the raw 14C DOC and d18O datasets in the Supplementary Material as it was not possible to examine the isotope data in detail.

A: We paste the 14C DOC age timeseries below. Note the inverse relation between F14C, plotted here, and DOC age. Decreasing 14C DOC age with time is evident for few wells only. For the anoxic well H53, the timeseries shows quite clearly that 14C DOC ages are decreasing and variance is increasing. For the others, it seems a little more difficult. We are planning to conduct a detailed investigation of the DOC 14C age timeseries once we gather more surrounding information to be able to provide a holistic intepretation. Within this study, we conclude that a) calculated similarities between soil seepage and groundwater DOM are generally well correlated to 14C DOC ages at the scale of considering all wells or all oxic wells, and b) calculated similarities seem more sensitive to surface-derived inputs for some wells (e.g. the suboxic H42 and H52, which show rather increasing 14C DOC ages with time). The raw 14C and stable isotope data are available in the R workspace linked in the code availability for people interested to inspect. We hope the reviewers accept this compromise as we still plan to investigate the 14C DOC data in more detail in the future.

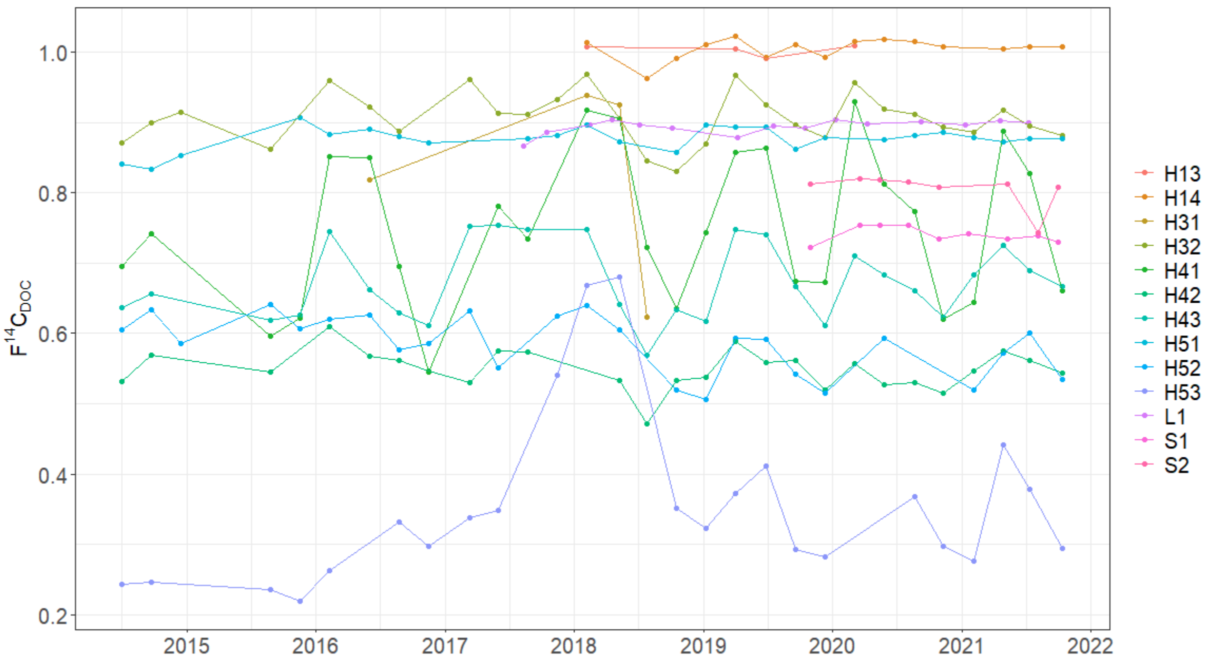

l. 105 – The authors write that this is a 'significant' increase. How is this significance determined?

A: We consider the observed effect sizes as significant changes in natural groundwater quality ($p<0.001$), because they strongly exceed a standard deviation of 0.05 percentage points based on the same calculations performed on repeated measurements (n=39) of an in-house groundwater DOM standard interspersed with the entire sequence of DOM DI-HR-MS measurements (Figure 2b).

l. 114 onwards – the authors argue that there is a change in composition after 2018, based on changeover values on linear regressions (Suppl Table 1). Could the authors clarify that this is this an analysis based on the data shown in Fig 2c? How are the changes in gradient tested statistically? Are they bigger than the uncertainty on the gradient?

A: There are no statistics in this case, because a) the linear regressions massively reduce the volume of available data to do statistics on and b) the expected variance between the wells is likely very heterogeneous. The slopes of the linear regressions, as shown in Suppl Table 1 are simply higher in many cases after 2018 than before 2018, which we think is an interesting observation to mention. To acknowledge the lack of statistics, we don't use the word „significant" here.

l.123 – It is noted here that max hydraulic heads were in 2017, although it looks like the highest hydraulic heads may have been early 2018 in the Hainich CZE on Supp Fig 1

A. True, this is now fixed. The respective sentences now reads: „During and following the summer of 2018, our investigation sites faced the greatest local annual soil water deficits within the period 2006-2021 (Supplementary Figure 5). These were accompanied by steep declines in hydraulic heads following previous groundwater highstands in early 2018 in the Hainich CZE (Supplementary Figure 1)."

l. 245 Methods – what bottles were used to store the stable water isotope, 14C and DOC/DOM samples?

A: We now specify in the methods that water was stored in 100 mL borosilcate bottles for DOC and stable isotopes, in 10 L HDPE canisters for SPE, and SPE eluates for HRMS and 14C in 10 mL

borosilicate vials.

L323 –Is *100 missing from the formula for percent similarity? I.e: 1−($Bray\ Curtis\ dissimilarity$) *100?

A: True, fixed.

The authors should specify the software/packages used to create all figures in each caption.

A: We added „Figure created in R.4.4.0 using package ggplot2." to all figure captions.

Andy Baker (UNSW) and Liza McDonough (ANSTO)

Reviewer #2 (Remarks to the Author):

I co-reviewed this manuscript with one of the reviewers who provided the listed reports. This is part of the Nature Communications initiative to facilitate training in peer review and to provide appropriate recognition for Early Career Researchers who co-review manuscripts.

Reviewer #2 (Remarks on code availability):

The code is available in the supplementary material and Github. It all looks to be complete however I wasn't able to install package "MFAssignR" to run the code for the raw data import.

A: Thanks, this is now fixed and MFAssignR should install properly.

Reviewer #3 (Remarks to the Author):

This paper discusses the relationship between groundwater levels, the concentration and composition of dissolved organic matter (DOM) in groundwater, and the source of this DOM in Europe between 2014-2021. The paper highlights the changes in observed groundwater levels and molecular DOM composition in response to hydroclimatic extremes, comparing these changes to the composition of DOM from surface (soil-seepage) derived sources. The authors conclude that hydroclimatic extreme events (i.e., periods of intense rainfall) promote the rapid movement of water and DOM between shallow surficial seepage and deeper groundwater aquifers at three Critical Zone sites, and that aquifer DOM is more similar to soil-seepage derived DOM following these hydroclimatic extreme events than under normal conditions. The authors conclude that this is because surface derived DOM moved through the aquifer unaltered by microbial processes that would otherwise degrade this DOM during its transport. In turn, the movement of unaltered DOM poses a concern for the safety of the aquifer drinking water resource, promoting aquifer redox conditions that may further contaminate the aquifer following hydroclimatic events. The authors discuss that the occurrence of these events is increasing, which in response, may exacerbate the deterioration of groundwater drinking water quality. The authors highlight the usefulness of DOM DI-HR-MS as a tool for detecting changes in groundwater deterioration and linking these changes to their source contributions. The authors also employ the KEGG database to predict relative abundances of metabolites that can be used to infer the metabolic functions of the groundwater microbiome. The study analyzed an impressive number of groundwater and soil seepage samples from three distinct critical zone research sites, including for DOC concentrations, stable hydrogen and oxygen isotope composition of the waters, radiocarbon of DOC, and DI-HR-MS. The study also predicted relative abundances of functional metabolites and microbial gene products recorded in 32 groundwater metagenomes.

Overall, I find this paper could be a valuable contribution to the scientific community. The paper is appropriate for Nature Communications and would be of interest to its broad reader base. Given the study’s use of data across space, time, and different types of measurements that each can provide source and process-based, mechanistic, understanding, I found that the authors need to expand the background, context, and elaborate on their findings in several areas of the paper. The authors share a concise story, but perhaps too concise given the complexity of the study. Nature Communications allows a generous word limit (5,000) that the authors should consider taking advantage of. By my estimate, the main body (not including Abstract, Methods, References, and Figure legends) is less than 1,000 words.

In my section and line by line comments below, I provide suggestions for the areas the authors could expand upon, with the goal of setting up a stronger background in the Introduction and elaborating on process-based and mechanistic linkages between the results and conclusions in the Results and Discussion.

A: Many thanks for encouraging us to expand and thus make our manuscript more accessible. We are aware that this study is written rather concisely and we hope the reviewer will accept our attempts to make a compromise between the scope we intended to cover in this investigation and giving sufficient background information.

Introduction

In Lines 33:35 the authors state that the “availability of valuable clean groundwater is threatened by

increasingly extreme hydroclimatic conditions that alter the functioning of natural recharging processes" but they do not explain what an extreme hydroclimatic condition is, why type they are referring to, nor how their increasing occurrence alters the function of natural recharging processes that may threaten groundwater quality. It's then difficult for the reader to interpret the study's main hypothesis in Line 38:40.
A: We expanded the section in question by adding the following: "Extreme hydroclimatic conditions such as extended drought periods or lasting heavy precipitation events are known to have severe and complicated consequences on the biogeochemical processinng, the generation of mobile matter, and the hydraulic structure in soils and the vasdose zone15,16. In sum, greater seepage volumes with greater matter loads are translocated more rapidly along preferential flow paths downwards, thereby escaping retention in soil and the vadose zone."

Likewise, here the connection to the mechanism is not being described. What are the natural filtering and transformative processes that render groundwater clean and safe to drink? Examples that relate to those processes that the results of this study inform would be helpful.
A: We tried to make this clearer in the following paragraph, where the most important examples (filtering via mineral sorption and transformation via microbial communities) are explained.

Lines 43:46 – The authors need to first explain what DI-HR-MS measures before going into how the measurement has helped scientists understand DOM's role in mediating key biogeochemical processes.

A: We now mention that DI-HR-MS provides molecular-level analyses of DOM composition.

Similar to my comment above, including specific examples would be helpful here. How has DI-HR-MS helped scientists understand sorption of surface-derived substances to minerals and their active transformation. What kind of substances and what are they transformed into?
A: We agree that substance class information was missing. Assigning substance classes from DI-HR-MS data is a highly debated topic, even though some do it regularly without much consideration. We ourselves are sceptical of compound class annotation based on DI-HR-MS data via the Van Krevelen Diagram and thus intentionally omitted discussing previous findings based on that technique. We offer a compromise in stating the most well-documented DOM compoud class level transformation, which is the shift of DOM rich in plant and soil-derived aromatics to microbial necromass.

Lines 46:48 – Why have studies suggested DOM DI-HR-MS data can be used as a proxy for the aquifer properties mentioned here? Please elaborate.
A: This sentence and using the word ‚proxy' was maybe misleading. We rephrased to: „Recent groundwater studies demonstrated that DOM DI-HR-MS data could inform about aquifer hydrogeology31, microbial community health23,32, and groundwater quality/recharge dynamics33."

Lines 48:49 – There is little prior work mentioned that gives context to why long-term and large-scale efforts aimed at exploiting DOM DI-HR-MS data to infer groundwater quality under variable and extreme conditions is specifically needed (i.e., as opposed to other types of DOM measurements).
A: Agreed, we broadened the scope of this sentence from DI-HR-MS specifically to any DOM molecular composition data. It now reads: „However, long-term and large-scale efforts aimed at exploiting DOM molecular composition data to infer groundwater quality under variable and extreme conditions need to be improved."

Lines 58:64 – This space has been used to give the reader details that are repeated in the Methods. It would be valuable to use this space to elaborate on the study's approach to addressing its research

question/hypothesis, the purpose behind the measurements/analysis, and how they are being used in concert.

A: Very good suggestion, we rephrased the entire paragraph accordingly. It now reads: „The overarching idea of this study is to leverage the extreme molecular complexity of DOM and provide a simple and scalable metric that is more sensitive towards changes in natural groundwater purification efficiency than the commonly used bulk concentration of groundwater DOC. We base our assessment of groundwater purification efficiency on the molecular-level Bray-Curtis similarities of groundwater and soil seepage DOM under changing hydroclimatic conditions between 2014 and 2021 in three geologically distinct Critical Zone research sites in Germany36,37. Support for the interpretation of changes in DOM composition are provided by groundwater stable isotope ratios and DOM radiocarbon isotope ratios, which track groundwater infiltration and the transport of 14C-young surface-derived substances, respectively. We omit common assumptions of indicative compound classes and instead link DOM to metabolic functions recorded in groundwater metagenomes. Resulting data strongly suggest that hydroclimatic variability causes fundamental changes to natural groundwater quality and its impact is likely to intensify global groundwater crises38,39."

Results and Discussion

Lines 77:84 – Please elaborate how the patterns or changes in stable water isotope results specifically indicate the conclusions stated in this section.

A: We rephrased this section, it now reads: "Stable isotope ratios of precipitation carry a seasonally varying signal into the aquifers, where many contributing water sources (temporal and spatial) accumulate and mix43 (Figure 1c). The likelihood of groundwater infiltration fluxes to be able to affect and sway the stable isotope ratio of the local groundwater bodies is expected to be greater following heavy short-term rainfall than light rainfall more spread out over the season."

Lines 91:97 – What additional context or reference to other studies can be used to justify that the 10%, 5% and 1% DOM composition similarity is a significant change for the timeframe evaluated, 2014-2021? DI-HR-MS provides a compositional analysis but there is no mention of the types of compounds that became more similar or different in groundwater in comparison to their abundance in surficial soil-seepage over the study's timeframe. With this much valuable information on source and biogeochemical signatures, how come the authors didn't consider applying a mixing model to estimate proportional contributions of soil-seepage DOM to the groundwater DOM pool over time?

A: These points are difficult to follow up on. As there were multiple points, we adress each individually:

„What additional context or reference to other studies can be used to justify that the 10%, 5% and 1% DOM composition similarity is a significant change for the timeframe evaluated, 2014-2021?"

Long-term investigations relying on DI-HR-MS of DOM are so far (to our knowledge) not available, even less considering the utilization of Bray-Curtis similarities. Using the similarity metric in the way we did only really makes sense in studies that have a great number samples available, which is commonly not the case. Considering that, we believe the most important point to demonstrate to the scientific community is that DOM DI-HR-MS data is far more scalable than we thought. This fact is demonstrated best by utilizing the long-term repeated injections as a reference for calculating significance, the way we did in Fig 2, because it emphasizes the great long-term stability of the Orbitrap MS detector.

„DI-HR-MS provides a compositional analysis but there is no mention of the types of compounds that became more similar or different in groundwater in comparison to their abundance in surficial soil-seepage over the study's timeframe." This is on purpose, for two reasons. 1) Most DI-HR-MS

investigations of infer types of compounds via the van Krevelen Diagram. We believe this approach is fundamentally problematic, because the information it provides is a) not very detailed and b) very insecure. 2) We already did it in Benk et al (2019) for the Hainich CZE. I'm the first author of both studies, changed my last name since then. The same trend of aromatics from soil being transformed to more saturated compounds indicative of a microbial origin has been shown many times already. We believe multi-omics predictions like our DOM and metagenomics far surpass the classical ‚type of compound' predictions both in molecular detail and prediction accuracy.
„How come the authors didn't consider applying a mixing model to estimate proportional contributions of soil-seepage DOM to the groundwater DOM pool over time" We believe this approach would have suffered from the fact that DI-HR-MS data of DOM is only semi-quantitative.

Lines 97:99 – Likewise, what specific changes in the DOM molecular compounds, where, and at what times provide evidence of an intensifying soil-to-groundwater connection, coupled to a reduction in efficiency of natural filtrations and purifications that occur during percolation from the vadose zone into the aquifers?
A: To be able to discuss ‚DOM molecular compounds', we would need to predict compound classes via the Van Krevelen Diagram. We believe these predictions are flawed and hope that the linkage of DOM to microbial community functions as shown in Fig 3 can provide an alternative to compound class predictions from DI-HR-MS DOM data in the future.

Lines 100:102 – Please elaborate on the results that led the authors to reach this conclusion.
A: The respective sentence was rephrased and more information was added. It now reads: " Groundwater in fracture-flow aquifer systems appeared markedly more vulnerable than groundwater in porous, unconsolidated sands, considering the rates of DOM composition change being an order of magnitude lower at the Linde site compared to Hainich CZE and SESO (Supplementary Table 1). This highlights the importance of preferential flow paths along fractures in the subsurface, allowing surface-derived substances to evade purification processes during rapid groundwater infiltration. The role of fractures in providing preferential flow paths is even more important for marine bedrock like those of the Hainich CZE because of their matrix tightness (low porosity and permeability)45."

Lines 104: 107 – What process-based understanding can the authors provide to explain why DOC concentration did not show significant changes during the observation period, and what are the implication of this finding?

A: We added: "It is likely that the previously shown great bioavailability of surface-derived DOC in groundwater contributed to the stability of groundwater DOC. The change in DOM composition would thus be expected to be driven rather by microbial metabolites and surface-derived substances, which are not readily biodegradable and not individually highly abundant."

Lines 107-113 – How does the 14C results reinforce the validity-based DOM assessments. Please be explicit and point to the patterns observed with reference to other studies that indicate this is true.
A: We clarified in the text that 14C results validated only the surface-sourcing of our observed trend in DOM composition, not DOM quality assessment in general. For context, we reference to our previous investigation of DOM composition and age from the Hainich CZE (Benk et al, 2019). The section now reads: „Measurements of the radioactive 14C isotope on the same groundwater DOM extracts analyzed via DI-HR-MS provided independent validation of the interpretation that changes in groundwater DOM composition were being driven by surface-derived inputs. We observed a positive correlation ($p < 0.001$) between the fraction of modern carbon (F14C) in groundwater DOM and the calculated similarities between groundwater and soil seepage HR-MS spectra (Supplementary Figure 4). The correlation was not heavily driven by low F14C values in the anoxic wells H52 and H53 and

was also significant ($p < 0.001$) when considering only oxic groundwater from the Hainich CZE. This finding underscored the potential of DOM DI-HR-MS data as meaningful proxies by which to accurately appraise the impact(s) of soil seepage-sourced contributions to groundwater ecosystems."

Could modern sources of C be introduced from a different pathway?
A: We are currently working on disentangling the various potential sources of young and old carbon in the Hainich CZE aquifers. It's too early in the project to speculate yet, though.

Lines 126:129 – Which substances? And how does this provide evidence that this DOM had not been processed microbially and had evaded Critical Zone filtration and purification processes?
A: We agree that this part was unclear. We rephrased to: „In a previous multi-year investigation of soil seepage at the Linde site it was found that following summer dryness, DOM was rich in plant-derived aromatic substances that were not yet microbially processed46." We hope that this makes the link to Fig 3, which reveals polycyclic aromatic hydrocarbon degratation as one of the most stongly affected metabolic pathways, clearer.

In the "Implications for groundwater ecosystems" section, are there additional linkages that can be made between microbially-mediated metabolic pathways and specific changes in DOM compound classes/composition before and following extreme hydroclimatic events that shed light onto the potential for contaminant release?
A: Linked to previous replies, we just don't believe in DOM compound classes the way they are commonly inferred from DI-HR-MS data. We agree with the reviewer that these connections could be highly valauable, but we think that they will need to rely on methods that can provide unambiguous structural elucidation.

Reviewer #3 (Remarks on code availability):

The authors provide the DOM processing code using R computing software, which appears it can be easily reproduced.

Reviewer #4 (Remarks to the Author):

Schroeter et al. present a comprehensive investigation of DOM composition, source, and dynamics in natural groundwater in relation to precipitation patterns in central Europe over 8 years. Most of the data is from one particular CZO in Germany, but two other central European CZOs are also included, although with much fewer observational data points.

The authors make a case that DI-HR-MS analyses of DOM can serve as a proxy for groundwater quality degradation, based on its ability to detect compounds related to microbial metabolic pathways involved in xenobiotics' degradation and biosynthesis of toxins and anti-biotics in natural groundwater. They also show how hydroclimatic extremes may compromise the filtration and purification effect that soils typically provide for precipitation water percolating through the soil, by promoting quick transport to groundwater, presumably through preferential flow, and thereby increasing the presence of surface-derived organic carbon (including xenobiotics) in groundwater, potentially leading to groundwater quality degradation.

The amount of observational data included in this study is impressive and I find the approach

generally sound and the results interesting. However, I think that the brevity of the text has somewhat limited the authors to deliver clear and substantiated support from the data to some of their statements and the message being conveyed is somewhat convoluted to me. For example, it took me several thorough readings of the manuscript to understand that the only metabolic pathways that were actually considered from the KEGGS database and linked to the DI-HR-MS data were those concerning xenobiotics and biosynthesized toxins and anitbiotics. There is also a lack of detail explaining how some of the calculations were made and the motivation for those with commentary on potential limitations, assumptions etc associated with the statistical and other data processing/comparisons/interpretations being conducted. I have tried to pinpoint some of these aspects in my detailed comments below.

I think this study is well conducted and the results should be of interest to the scientific community, but I cannot be sure, because the text is not providing enough detail and focus to fully assess the quality of the study and relevance of results. This, together with the somewhat narrow scope of "only" including xenobiotics and biosynthesized toxins and antibiotics (which definitely should be made clear very early on to avoid misunderstandings of what "metabolic pathways" are considered in this study) puts this slightly outside of what I would consider suitable for Nature Communications. I think it may fit better in a slightly less broad scientific journal. However, if edits were made to address these concerns, it may well be that this manuscript could be suitable for Nature Communications, especially if a bit more effort was put towards clarifying how the broader statements being made are supported by the data and also put into a context of the importance of xenobiotics and biosynthesized toxins and antibiotics for groundwater quality in more concrete terms.

A: Many thanks for providing valuable feedback our manuscript. In accordance with your and other reviewer's comments, we expanded among others on methods description and discussion of our results in various sections throughout the manuscript.

Detailed comments

L26 Suggest changing "naturally occurring xenobiotic substances" to "xenobiotic substances occurring in natural groundwater" as xenobiotics by definition are not "natural". Also, the biosynthesized antibiotics and toxins should not be considered "xenobiotics", and "xenobiotics" cannot be biosynthesized (then they would not be "xenobiotic"). This is also not completely clear in other parts of the manuscript (e.g., L217 in Fig. 3 caption)

A: Thanks for the suggestion. L26 was adapted accordingly. The pathway group „Xenobiotics biodegradation and biosynthesis" is a fixed term within the KEGG database and contains a specific set of pathways. We adapted the caption of Fig. 3 to make this distinction clearer. It now reads: „…Pathways belonging to the KEGG pathway group 'Xenobiotics biodegradation and biosynthesis' are colored in red. Other pathways relating to the biosynthesis of natural antibiotics and toxins as well as abundant pathways are named in black text."

L54-55 Suggest refraining from assertively stating that the study is "the most comprehensive" unless you have actual concrete proof of it – in which case this should be cited or at least supported by how you arrived at this conclusion. Better to state "one of the most comprehensive" or similar.

A: Done as suggested: „…one of the most comprehensive…“

L114 (and supplementary Table 1, see comment below) It is not clear to me how the year-on-year changeover is calculated.

A: We now mention that the values represent slopes of linear regressions. The sentence in question now reads: „The rate of year-on-year changeover in groundwater DOM composition was greater following the extreme summer drought of 2018 compared to the preceding period, based on respective linear regressions of the similarity values shown in Figure 2c (Supplementary Table 1).“

L120-121 “anthropogenic warming” sounds like it is intentional warming (e.g., as part of an experiment), suggest changing to “anthropogenic-induced global warming”

A: Changed accordingly to „…anthropogenic-induced global warming…“

L142 (or somewhere else in this section) Suggest specifying that the targeted metabolic pathways were related to “xenobiotic degradation” and “natural toxin and antibiotic biosynthesis”, assuming that this is indeed the case based on results presented.

A: Our analysis was not targeted towards any pathways except for the requirement of matching pathways between DOM and metagenomes. The highlighting of pathways related to xenobiotics and antibiotics/toxins is data-driven. We plotted all the predicted pathways in Fig 3, and these had some of the highest correlation scores and abundances. That's why we highlight them and discuss them in the text. All other pathways, that are not mentioned in the text, but are shown as dots in Figure 3 have their data available in the workspace image, which is linked to the supplementary R code.

Fig. 1 The Hainich (blue) text is blurry to the extent that it is difficult to look at the figure. I understand there are a lot of points, but would be good if text could be sharper.

A: Unfortunately, upon embedding into the manuscript and rendering as PDF, image quality suffered a lot. We have made the original vector graphics available in the review forum. These are perfectly sharp. We hope the editing team will embed the vector graphics for the final version better than we can do now. The reviewer can inspect the vector graphics (.svg files) for example with the free software Inkscape. In accordance with other reviewer's comments, Fig 1a was updated to show points instead of text to make the figure clearer and the color scheme for the Linde site was updated to improve visibility.*

Fig. 2 I find “decomposition” to be confusing in the figure title – what is meant by this term here? Also, the sentence on L201-202 is not clear and the explanation in the methods section (L323-325) does not fully clarify what samples were compared to each other (all sites at all times? By site? How were the temporal trends assessed in that case?). Please provide more specifics to help clarify what was done (can go in Supplementary Information if needed)

A: Good point by the reviewer. We added more information to the method section: „PS values for groundwater from the Linde site reference to soil seepage from Linde, those for groundwater from Hainich CZE and SESO reference to soil seepage from Hainich CZE. PS values were first calculated between all samples, then averaged over all soil seepage sampling time points and local sampling locations to maximize robustness against surface heterogeneity.“
Regarding the title of Figure 2 we're having a hard time to make changes, beacuse seasonal time series decomposition is a very common method and therefore standard terminology in time series analysis. Keeping this exact wording will enable the readers to very quickly look up the method and

find many detailed explanations and tutorials on this method online.

Fig. 3 Suggest referring to Supplemental Figure 6 that explains the workflow for connecting DI-HR-MS data to KEGG. It is also not clear to me how the 254 DOM samples connect to the 32 genome samples. Are all DOM data for a specific sampling location (independent of time point) compared to a single groundwater genome data point? And were the groundwater genome data generated from any of the samples used for DOM analyses or were these from previous samples that had been collected and sequenced at an earlier stage (this is somewhat implied on L347)? The current writing in both the manuscript and the methods section are ambiguous. It is important to understand the relationship between the samples that were used for the two types of analyses in order to evaluate the relevance of data correlations.

A: We extended the caption of Figure 3 with the following: „Detailed information on the pathway prediction workflow is available in Supplementary Figure 6.“ In addition, we now clarify in the methods section that sampling for metagenome sequencing took place in January 2019 and that metabolic pathway predictions from DOM were filtered to match predictions from metagenomes of the same respective well.

Throughout Methods section – For all instruments, please add models and manufacturer locations

A: Updated accordingly.

L272-273 Suggest adding a bit more detail about the lysimeters, although the information is provided in the cited publications. For example, although the lysimeters installed at these sites are indeed the "original" kind – i.e. porous plates placed underneath a soil-filled cylinder, recent use of the term has been applied to other types of suction pore water samplers, so it would be prudent to specify the type used here. It would also be nice as a reader to get the dimensions, pore size and material of the porous plate, and collection depth(s) without needing to look up the citations. Also, for Hainich – if I understand correctly) lysimeters are installed at 2 depths – were samples collected from both or only at the topsoil/subsoil interface?

A. Good point. All available depths were samples. We added the following information on the specifications of the soil seepage sampling to the method section: „Details on the setup of the lysimeters in mixed beech forest and grassland in Hainich CZE are available in Lehmann et al., 202134. Briefly, lysimeters in Hainich CZE were set up in 30 and 60 cm depth and featured porous silicon carbide suction plates (SIC320, mean pore size: 21 µm, thickness 10 mm, area 0.08 m²; METER, Vöhringen, Germany) installed below an undisturbed soil profile without sidewalls. Details of the setup in Beech, Oak, and Pine forest as well as grassland at the Research Station Linde are available in Orme et al., 202246. Briefly, sintered glass suction plates (pore size: 1-1.6 µm, thickness: 10 mm, area 0.045 m²; UMS, Willmars, Germany) were installed in Linde in 5, 10, 20 and 30 cm depth below an undisturbed soil profile.“

L 326 What is meant "by loess" here?

A: We capitalized LOESS and added in brackets: „locally estimated scatterplot smoothing".

L329 Why was P not considered in the formula assignments?

A: We did assign P in a previous version of this manuscript and one reviewer at the time argued that P could not be be reliably assigned to DOM sum formulas and doing so would majorly impact the

results. Thus, we re-ran all analyses without assigning P. The results remained largely unaffected.

Supplementary Information

Supplementary Figure 7 Very minor, but the percentage of unambiguous pathways predictions was 72% in Supplementary Figure 6 (which matches the numbers shown in that figure), so just wondering why it would be stated as 73% in the caption here.

A: Thanks for catching this, changed to 72%.

Supplementary Table 1 I am confused by what is meant by “Year-on-year changeover” when in the next sentence it is stated that linear regressions were calculated for data before and after July 2018 (i.e. multiple years for each regression). It makes sense to divide the time series this way based on the 2018 drought induced shift, but I am struggling to understand how the year-on-year changeover similarities is calculated. Also, it is not clear to me what the numbers presented in the table actually represent. What does pp stand for in the units used?

A: We agree that this was unclear. The values are simply the slopes oft he linear regressions and the units are percentage points per year, because the similarities are already given in percent. The caption of Supplementary Table 1 now reads: „Year-on-year changeover in similarities between groundwater and soil seepage DOM HR-MS spectra. Values represent the slopes of linear regressions of the percentage similarity values presented in Figure 2, split into before and after July 2018, the height of the extreme summer drought in Europe. Slopes of the linear regressions are given in percentage points per year (pp/a). Missing values (NA) indicate insufficient data during the respective period.“

Reviewer #4 (Remarks on code availability):

I didn't see any code included i the supplementary materials

A: Code was uploaded as a separate file It must be there somewhere as other reviewers did comment on it.

Reviewer #1 (Remarks to the Author):

This is again a co-review by Andy Baker (UNSW) and Liza McDonough (ANSTO).

We thank the authors for their thorough response to the reviewers, and the updated code.

We (that is, Andy) have only one question remaining which relates to Figure 1b. Thank you for confirming the normality of this data, and that the analysis is on calendar years. We suggest the latter information is included in the figure caption? However, I am still not convinced that the statistic provided is robust. For example, I couldn't see that the question of temporal autocorrelation was addressed. If the individual time series are temporally autocorrelated, this will affect the significance of the correlation coefficient. Also, the y-error is 0.1 per mille on each analysis, but is this included in the regression analysis? My original concern remains - the analytical uncertainty in each data point is greater than the trend in this data, the r value is not that strong, and the data from all the bores has been combined in order to produce this statistic. This reviewer (Andy) would have greater confidence in this analysis if it was demonstrated to reproducible e.g. on a repeat analysis where hydrological years were used, and/or a repeat analysis where the y-error was included and/or Kendall's tau was used.

A1: As there are two major points (correlation and y-error), we answer this question in two parts. We followed the reviewers suggesion and changed the regression method to Kendall's tau. The resluting coefficient is highly similar to the previously obtained Pearson's R. Both methods demonstrate a highly significant positive relationship between the annual variances in groundwater delta18O over the observed period ($p<0.001$). We updated Figure 2 and its caption accordingly. Please find below exemplary code and results on Pearons R vs Kendalls tau that can be tested in the supplementary R workspace. The second part of our answer follows below the code section.

Code

```
> cor.test(var %>% filter(`aquifer type` == "Hainich CZE") %>% pull(variance),
+         var %>% filter(`aquifer type` == "Hainich CZE") %>% pull(year))
```

Result

```
Pearson's product-moment correlation

data:  var %>% filter(`aquifer type` == "Hainich CZE") %>% pull(variance) and var %>% filter(`aquifer type` == "Hainich CZE") %>% pull(year)

t = 5.4568, df = 71, p-value = 6.71e-07

alternative hypothesis: true correlation is not equal to 0

95 percent confidence interval:

 0.3583147 0.6876437

sample estimates:

      cor

0.5435718
```

Code

```
> cor.test(var %>% filter(`aquifer type` == "Hainich CZE") %>% pull(variance),
+         var %>% filter(`aquifer type` == "Hainich CZE") %>% pull(year),
+         method = "kendall")
```

Result

```
Kendall's rank correlation tau

data:  var %>% filter(`aquifer type` == "Hainich CZE") %>% pull(variance) and var %>% filter(`aquifer type` == "Hainich CZE") %>% pull(year)

z = 6.4096, p-value = 1.459e-10

alternative hypothesis: true tau is not equal to 0

sample estimates:

      tau

0.5390949
```

A2: We provide below a plot of the average absolute deviations from the annual mean delta18O. It is important to differenciate between the deviation from the mean and the variance. These two share the following relation: $var = \sum(x - \bar{x})^2/(n - 1)$ with $(x - \bar{x})$ as the deviation from the mean, *var* as the variance, and *n* as the number of observations. The plot below shows that the average absolute deviations from the annual mean delta18O per well and year range from around 0.1 permil to up to 0.3 permil with a significantly positive trend over time. The reviewer correctly notes that the deviations from the mean between 2014 annd 2017 were within the range of the measurement error of 0.1 permil. Between 2018 and 2021 the deviations from the mean grow to up to triple that value. The observed effect sizes thus indeed exceed measurement error. The values of annual variances reported in figure 1b are lower only because of the necessary division by (n-1) and do not reflect issues with effect size. We report variances in the manuscript instead of deviations from the mean, because the number of observations per well and year varies a bit and dividing by the number of observations accounts for that.

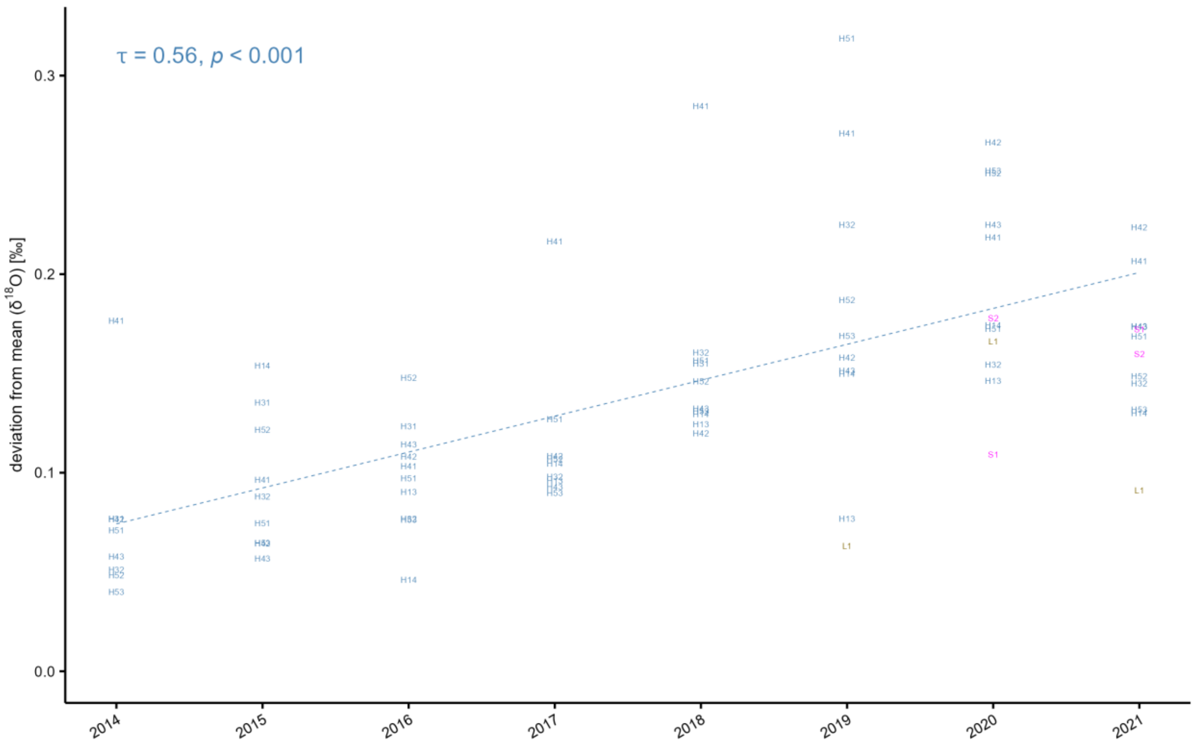

Reviewer #1 (Remarks on code availability):

Thank you for the revised code which now seems to work and contains the 14C and d18O data. I noticed that I had to add Library(broom) to the top of the code in order to run the piece of code titled #LINEAR REGRESSIONS OF SIMILARITIES TO SOIL SEEPAGE DOM. It is suggested that this library gets added to the supplementary code prior to final upload.

A: Done as suggested.

Reviewer #2 (Remarks to the Author):

I co-reviewed this manuscript with one of the reviewers who provided the listed reports. This is part of the Nature Communications initiative to facilitate training in peer review and to provide appropriate recognition for Early Career Researchers who co-review manuscripts.

Reviewer #2 (Remarks on code availability):

See comments from co-reviewer.

Reviewer #3 (Remarks to the Author):

The authors seemed to have thoughtfully considered the reviewer suggestions and made subsequent changes to address the concerns raised in the reviewer comments. I find that the changes made improve the paper overall.

In their response letter, the authors explain why they chose not to use the DI-HR-MS DOM composition data to infer types of compound classes via the van Krevelen Diagram, and in turn use similarities, differences, and/or changes in the relative abundances of these compound classes in the study's analysis, as most studies do. The authors state that the study's multi-omics prediction approach, linking DOM composition to microbial community functions, far surpass this type of compound predictions both in molecular detail and prediction accuracy, and thus provide an improved alternative to compound class predictions from DI-HR-MS DOM data. Since compound classification of DI-HR-MS DOM composition data is a commonly applied technique, the argument against inferring compound classes via the van Krevelen Diagram and the reasons for implementing the alternative approach as done in the study and in the future should be discussed in greater detail in the paper.

A: Here, we propose the following alternative formulation, as we believe that metabolic pathway predictions from our multi-omics approach go beyond compound class predictions based on the Van Krevelen diagram and offer a more detailed insight.

„Linking DOM and metagenomes provided insights beyond those achieved with traditional methods. More than forty distinct metabolic pathways were jointly predicted by DI-HR-MS and metagenomics data. In a previous investigation of DOM at the Hainich CZE, we evaluated four compound classes based on the Van Krevelen Diagram: polyphenols, highly unsaturated substances, unsaturated aliphatics, and peptide-like substances[35]. These structural features not only lacked a precise functional description but have since been shown to loosely overlap with their respective regions in the Van Krevelen Diagram[80]. Here, more than 70% of sum formulas could be unambiguously linked to specific metabolic pathways, largely due to the requirement for matching predictions based on metagenomes."

Reviewer #4 (Remarks to the Author):

The manuscript has been greatly improved and my concerns have been met. I apologize to the authors for missing some additional material in the first round.
